# SlS5H silencing reveals specific pathogen-triggered salicylic acid metabolism in tomato

**DOI:** 10.1186/s12870-022-03939-5

**Published:** 2022-11-29

**Authors:** C. Payá, S. Minguillón, M. Hernández, S. M. Miguel, L. Campos, I. Rodrigo, J. M. Bellés, M. P. López-Gresa, P. Lisón

**Affiliations:** Instituto de Biología Molecular y Celular de Plantas (IBMCP), Consejo Superior de Investigaciones Científicas (CSIC), Universitat Politècnica de València (UPV), Ciudad Politécnica de la Innovación (CPI) 8 E, Ingeniero Fausto Elio s/n, 46011 Valencia, Spain

**Keywords:** Defence, Metabolomics, Pathogen, Phenolics, Plant stress, Salicylic acid, Tomato

## Abstract

**Background:**

Salicylic acid (SA) is a major plant hormone that mediates the defence pathway against pathogens. SA accumulates in highly variable amounts depending on the plant-pathogen system, and several enzyme activities participate in the restoration of its levels. Gentisic acid (GA) is the product of the 5-hydroxylation of SA, which is catalysed by S5H, an enzyme activity regarded as a major player in SA homeostasis. GA accumulates at high levels in tomato plants infected by Citrus Exocortis Viroid (CEVd), and to a lesser extend upon *Pseudomonas syringae* DC3000 pv. *tomato* (*Pst*) infection.

**Results:**

We have studied the induction of tomato *SlS5H* gene by different pathogens, and its expression correlates with the accumulation of GA. Transient over-expression of *SlS5H* in *Nicotiana benthamiana* confirmed that SA is processed by SlS5H in vivo. *SlS5H*-silenced tomato plants were generated, displaying a smaller size and early senescence, together with hypersusceptibility to the necrotrophic fungus *Botrytis cinerea*. In contrast, these transgenic lines exhibited an increased defence response and resistance to both CEVd and *Pst* infections. Alternative SA processing appears to occur for each specific pathogenic interaction to cope with SA levels. In *SlS5H*-silenced plants infected with CEVd, glycosylated SA was the most discriminant metabolite found. Instead, in *Pst*-infected transgenic plants, SA appeared to be rerouted to other phenolics such as feruloyldopamine, feruloylquinic acid, feruloylgalactarate and 2-hydroxyglutarate.

**Conclusion:**

Using *SlS5H*-silenced plants as a tool to unbalance SA levels, we have studied the re-routing of SA upon CEVd and *Pst* infections and found that, despite the common origin and role for SA in plant pathogenesis, there appear to be different pathogen-specific, alternate homeostasis pathways.

**Supplementary Information:**

The online version contains supplementary material available at 10.1186/s12870-022-03939-5.

## Background

Salicylic acid (SA or 2-hydroxy benzoic acid) is a phenolic compound present in many plants, and involved in different physiological and biochemical processes, being the activation of inducible defence programs its best characterized function. SA was first described to act in tobacco as an inducer of plant disease resistance to tobacco mosaic virus [1]. Subsequently, evidence suggesting SA is a signal molecule comes from the landmark studies in tobacco mosaic virus-resistant tobacco and cucumber upon infection with necrotizing pathogens [2, 3]. The essential role of SA in plant defence was definitively demonstrated by using transgenic tobacco plants unable to accumulate SA, which resulted to be incapable to establish the well-known systemic acquired resistance (SAR), an induced defence that confers long-lasting protection against a broad spectrum of pathogens [4, 5]. To date, many other studies have been published to point out SA as the best known defence-related hormone [6, 7].

This phenolic compound is biosynthesized in plants from phenylalanine through the route of the phenylpropanoids (PAL pathway) or from isochorismate (IC pathway). Loss-of-function of some genes from both pathways results in an increased plant susceptibility to pathogens, indicating that both the IC and PAL pathways contribute to SA accumulation and function in response to biotic stresses. Nevertheless, the main source of SA when the plant faces a pathogenic infection and SAR is established mostly depends on the IC pathway in *Arabidopsis thaliana* [8, 9], being the pathway downstream of IC completely deciphered [10, 11]. In this sense, upon stress situations, ICS1 and ICS2 isochorismate synthase activities isomerise chorismate into IC. In plants, IC is conjugated to the amino acid L-glutamate by an isochorismoyl-9-glutamate (IC-9-Glu) that can spontaneously break down into SA. Besides, EPS1 (Enhanced Pseudomonas Susceptibility 1) is an IC-9-Glu pyruvoyl-glutamate lyase that can enhance the conversion of IC-9-Glu into SA more effectively [12].

Due to its cytotoxic effects, plants maintain SA homeostasis by fine-tuning the balance between the biosynthesis and catabolism of this phytohormone. In this way, SA can be chemically modified into different bio-active derivatives, through glycosylation, methylation, sulfonation, amino acid conjugation, and hydroxylation [7]. Most of the SA present in the plant is glycosylated into SA 2-*O*-β-D-glucoside (SAG) [2, 13] and, to a lesser extent, into salicylate glucose ester (SGE) [14], being both stored in the vacuole. These conjugates constitute a reserve of inactive SA that can be released slowly in its active form when the plant needs it by the action of glucosyl hydrolases [15, 16]. In addition to this, SA can be conjugated with amino acids such as aspartic acid into SA-Asp [17], converted into SA-2-sulfonate by sulfotransferases [18], or methylated to form methyl salicylate (MeSA) by salicylic acid carboxyl methyltransferase (SAMT), this latter modification increasing SA membrane permeability and facilitating their mobilization. In SAR, MeSA acts as a phloem-based mobile signal that, after its hydrolysis to SA, triggers resistance [19, 20].

Regarding SA hydroxylation, a salicylate 3-hydroxylase (AtS3H) was described in Arabidopsis. This hydroxylase is induced by SA and converts this compound into both 2,5-dihydroxybenzoic acid (2,5-DHBA) and 2,3-dihydroxybenzoic acid (2,3-DHBA) in vitro, and only into 2,3-DHBA in vivo. Studies with *s3h* mutants and the gain-of-function lines revealed that S3H regulates Arabidopsis leaf longevity by mediating SA catabolism [21]. DMR6 (Downy Mildew Resistant6) oxygenase has been proven essential in plant immunity of Arabidopsis [22] and has been later described as a salicylic acid 5-hydroxylase (AtS5H) that catalyses the formation of 2,5-DHBA displaying higher catalytic efficiency than S3H. The Arabidopsis *s5h* mutants and *s5hs3h* double mutants over accumulate SA and display phenotypes such as a smaller size, early senescence, and enhanced resistance to *Pseudomonas syringae* pv. *tomato* DC3000 [23]. More recently, the tomato DMR6 orthologs *SlDMR6-1* and *SlDMR6-2* have been identified, also displaying SA 5-hydroxylase activity. Tomato *SlDMR6-1* mutants, obtained by CRISPR/Cas9 system, exhibited broad spectrum disease resistance, correlating this resistance with increased SA levels and transcriptional activation of immune response upon *Xanthomonas gardneri* infection [24]. Therefore, preventing SA hydroxylation confers resistance to pathogens both in Arabidopsis and tomato, standing 2,3-DHBA and 2,5-DHBA for deactivated forms of SA. The main function of these hydroxylated forms is to prevent SA from over-accumulating, thus constituting a mechanism by which plants fine-tune SA homeostasis [21, 23–25].

The 2,5-DHBA or gentisic acid (GA) has been described in animal tissues [26], in microorganisms [27], and plants [28]. Similar to SA, GA accumulates as glycoconjugates in plants, primarily as GA 5-*O*-β-D-glucosides, GA 5-*O*-β-D-xylosides, or GA 2-*O*-β-D-xylosides [29–32]. It is also known that exogenous application of GA to tomato (*Solanum lycopersicum*), cucumber (*Cucumis sativus*), and *Gynura aurantiaca* induces the expression of a distinct subset of PR defensive genes compared with the genes induced by SA, as well as mechanisms of gene silencing, and plant resistance [33–35].

The accumulation of SA and GA varies up to 100-fold in different plant species [23]. In tomato, that range of difference also occurs among different types of infections, being the accumulation of GA much higher than the SA itself in all of them [33, 36–38]. These differences raise the hypothesis that plants regulate SA homeostasis differently upon specific pathogenic attacks, instead of displaying common pathways to metabolise SA. Since the conversion of SA into GA appears to play an essential role in SA homeostasis, our objective was to delve into the defensive role of a tomato salicylic acid 5-hydroxylase, and our results provide new insights into the SA metabolism of tomato plants facing different infections.

## Results

### Identification of the tomato pathogen-induced ortholog of *Arabidopsis thaliana S5H*

To identify the enzyme responsible for the conversion of SA into GA in tomato, a Blastp analysis was performed in Sol Genomics databases (https://solgenomics.net/) by using the *AtS5H/DMR6* sequence (At5g24530). A phylogenetic tree was built including the closest tomato sequences (Supplemental Fig. S1). The Solyc03g080190 was selected as a candidate for SH5 role in tomato, since it resulted to be one of the closest in the phylogenetic tree, and it presented the highest identity percentage (67.35%), in contrast to the 62.43% of identity displayed by the Solyc06g073080 sequence, also near in the phylogenetic tree. The Blastp analysis of the Solyc03g080190 in The Arabidopsis Information Resource (www.arabidopsis.org) confirmed the selected candidate, being *AtS5H/DMR6* the closest gene to Solyc03g080190 *(SlS5H)* in *Arabidopsis thaliana*. Finally, this sequence coincided with At5g24530 tomato ortholog proposed by *EnsemblePlants* (http://plants.ensembl.org/index.html) and with *SlDMR6-1*, which has been recently proposed as the *DMR6* ortholog in tomato and which displayed salicylic acid 5-hydroxylase activity [24].

**Fig. 1:**
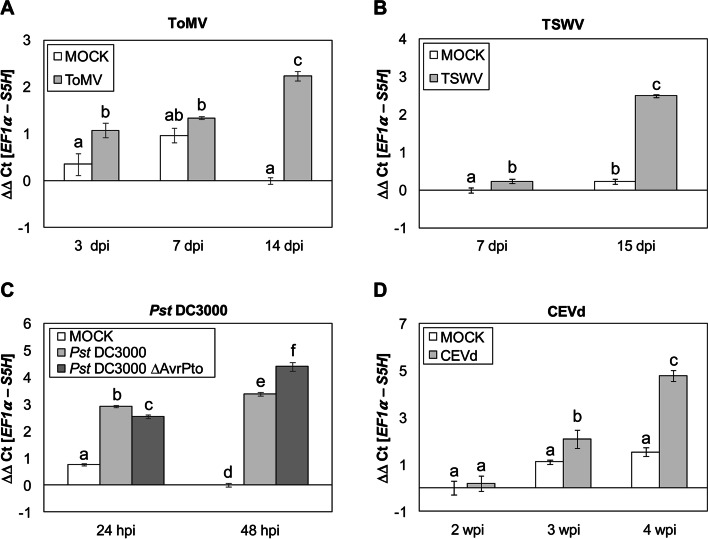
*SlS5H* expression patterns induced by different pathogens. Expression of *SlS5H* gene after inoculation with Tomato Mosaic Virus (ToMV, **A**); Tomato Spotted Wilt Virus (TSWV, **B**); *Pseudomonas syringae* pv. *tomato* DC3000 (*Pst*DC3000 **C**) and Citrus Exocortis Viroid (CEVd, **D**) at different times post-inoculation. MOCK represents the mock-inoculated plants. The qRT-PCR values were normalized with the level of expression of the elongation factor 1 gene. The data are presented as means ± standard deviation of a representative experiment (*n* = 3). An ANOVA test was performed and statistically significant differences (*p*-value < 0.05) between infected or mock-treated plants at different time points are represented by different letters

According to available transcriptome data, *SlDMR6-1* expression has been described to be induced in response to several pathogens [24]. To confirm the pathogen triggered *SlS5H* induction, and to extend the study of expression to other pathogens which provoke the accumulation of SA and GA, tomato plants were subjected to infection either with Citrus Exocortis Viroid (CEVd), Tomato Spotted Wilt Virus (*TSWV*), Tomato Mosaic Virus (*ToMV*) or a virulent and an avirulent strain of *Pseudomonas syringae* pv. *tomato* DC3000 (*Pst*) (see Materials and Methods). Leaf samples were collected at the indicated time points and *SlS5H* expression levels were analysed by qRT-PCR in those tomato-pathogen interactions (Fig. 1 A to D). The induction of *SlS5H* was observed upon all the pathogen infections in the analysed samples, reaching levels up to 5 times higher in CEVd-infected or *Pst-*infected plants than those observed in the non-infected plants, and around 2 times higher in the case of TSWV or ToMV infections. It is worthy to note that these induction patterns correlated with symptomatology, producing infection with CEVd the most severe disease symptoms and being ToMV infection practically symptomless [39]. Regarding the bacterial infection, *SlS5H* expression levels were higher in the tomato plants inoculated with the virulent bacteria (*Pst* DC3000 ΔAvrPto) as compared to the avirulent infection at 48 h post inoculation (hpi), therefore confirming this tendency.

To study the induction of *SlS5H* by its own substrate, SA treatments were performed, and samples were collected at different time points (see Materials and Methods). As Fig. S2A shows, a statistically significant induction of *SlS5H* was detected by qRT-PCR at 6 h post treatment (hpt) when compared with non-treated plants, presenting the maximal induction at 1 hpt. *PR1* activation was used as a positive control for the treatments, presenting a significant induction at 24 hpt (Fig. S2B).

All these data confirm that *SlS5H* is involved in the plant response to pathogens, extending its putative role to different tomato-pathogen interactions.

### Overexpression of *Sl5H* in* Nicotiana benthamiana *decreases SA levels *in vivo*

To confirm the S5H biochemical activity in vivo, *Nicotiana benthamiana* plants were agroinoculated with a construction carrying the *SlS5H* cDNA containing a His tag under the *35S CaMV* promoter. These plants (*pGWB8-SlS5H*) and the corresponding control plants inoculated with the empty plasmid (*pGWB8*) were then embedded with SA (see Materials and Methods). The accumulation of the recombinant protein was confirmed by western blot analysis in *pGWB8-SlS5H* plants, and levels of free and total SA were measured (Fig. S3). As expected, levels of free and total SA were almost 3 times lower in *pGWB8-SlS5H* plants, being these differences statistically significant, and thus confirming that SA is a substrate for SlS5H in vivo. However, no differences in neither the GA nor in 2,3-DHBA accumulation were detected between pGWB8 and pGWB8-SlS5H *Nicotiana benthamiana* plants (Fig. S3D).

### Silencing *SlS5H* increases resistance to CEVd in tomato

To gain further insights into the in vivo role of SlS5H, silenced transgenic Moneymaker tomato plants were generated by following an RNAi strategy (see Materials and Methods). The generated tomato lines *RNAi_SlS5H* were characterized, and several independent transgenic lines were confirmed. Homozygous lines *RNAi_SlS5H 14* and *RNAi_SlS5H 16* both carrying one copy of the transgene, were selected for further studies.

**Fig. 2:**
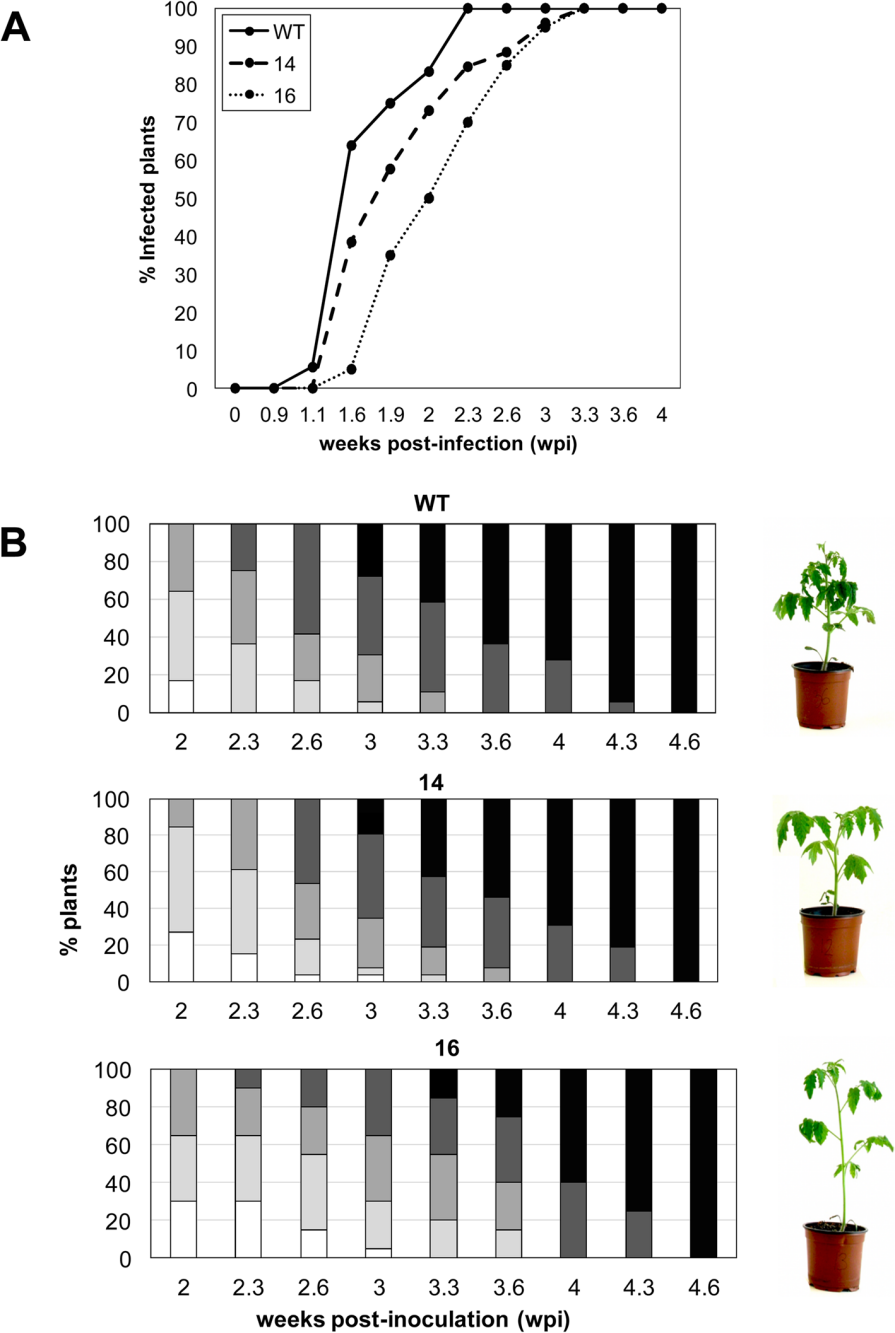
Symptomatology of wild type (WT) and *RNAi_SlS5H* (lines 14 and 16) tomato plants infected with CEVd. **A** Disease development in WT and *RNAi_SlS5H 14* and *RNAi_SlS5H 16* plants infected with CEVd. Evolution of the percentage of tomato plants showing symptoms at the indicated time points (wpi). **B** Representative images and disease severity of CEVd infected WT and *RNAi_SlS5H* plants. Symptomatology was established using the following scale: no symptoms (white), mild epinasty (light grey), severe epinasty and stunting (grey), leaf rugosity (dark grey), midvein necrosis and chlorosis (black). Data correspond to one representative experiment

To extend the role of *SlS5H* in plant defence, the tomato-CEVd interaction was selected, since GA -the proposed product of S5H activity- had been described to accumulate at very high levels in CEVd-infected tomato plants [33, 36–38]. Therefore, wild type (WT) and *RNAi_SlS5H* transgenic plants were inoculated with CEVd and checked for the development of symptoms. The characteristic symptomatology of CEVd-infected tomato plants consists of epinasty, stunting, leaf rugosity, midvein necrosis and chlorosis [40]. As Fig. 2A shows, differences in the percentage of plants displaying symptoms were observed in both *RNAi_SlS5H* transgenic lines with respect to the parental tomato plants. Particularly, transgenic line *RNAi_SlS5H 16* displayed only 35% of plants showing symptoms at 1.9 weeks post inoculation (wpi), while almost 75% of non-transgenic plants exhibited them. Moreover, at 2.3 wpi all the WT plants displayed symptoms while around 20% of the *RNAi_SlS5H* remained symptomless. These results indicate a delay in symptom appearance in CEVd-infected *RNAi_SlS5H* tomato plants.

To confirm the differences in the disease development observed, a scale of the disease severity was established, scoring symptoms from mild (mild epinasty) to very severe (midvein necrosis and chlorosis), at different time points (see Materials and Methods). As Fig. 2B shows, differences were observed between WT and *RNAi_SlS5H* transgenic tomato plants from 2.3 to 3.6 wpi. Moreover, *RNAi_SlS5H 16* transgenic line did not display very severe symptoms at 3 wpi, whilst 30% parental plants exhibited severe symptoms at the same time point. Therefore, the observed differences in symptom severity confirmed the partial reduction in the susceptibility of *RNAi_SlS5H* tomato plants to CEVd infection. Interestingly, transgenic plants appeared to display a higher internode length when compared to the control plants. This phenotype was quantified as significant in further studies (Fig. S6B).

**Fig. 3:**
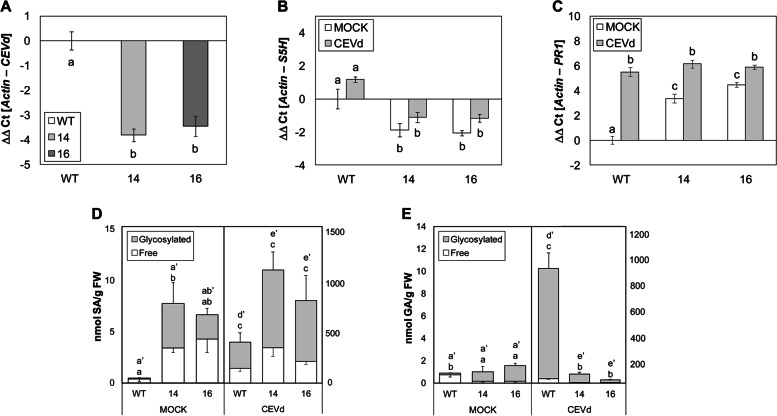
Characterization of wild type (WT) and transgenic *RNAi_SlS5H* (lines 14 and 16) tomato plants infected with Citrus Exocortis Viroid (CEVd). Tomato plants were inoculated with CEVd. Leaf samples were taken 3 weeks after inoculation and analyzed for *CEVd* (**A**), *S5H* (**B**) and *PR1* (**C**) gene expression, and phenolic compound accumulation (Salicylic Acid, SA, **D**; Gentisic Acid, GA, **E**). MOCK represents the non-inoculated plants. The qRT-PCR values were normalized with the level of expression of the actin gene. The data are presented as means ± standard deviation of a representative experiment (n > 3). Statistically significant differences (*p*-value < 0.05) between genotypes and infected or mock-treated plants are represented by different letters. Significant differences in SA and GA accumulation are analysed in free (x) and total (x’) phenolic compounds

Finally, to confirm the enhanced resistance, the presence of pathogen was measured at 3 weeks post-inoculation (wpi), detecting a statistically significant decrease in the CEVd accumulation in both *RNAi_SlS5H* transgenic lines (Fig. 3A).

Our results appear to indicate *S5H* silencing reduces tomato susceptibility to CEVd, confirming the role of *SlS5H* in the plant defence response.

### Silencing *SlS5H* causes an activation of plant defence upon CEVd infection

To analyse *SlS5H* expression levels in the RNAi transgenic plants infected with CEVd, qRT-PCR from WT, *RNAi_SlS5H 14* and *RNAi_SlS5H 16* plants, were performed at 2 weeks after the inoculation with CEVd (Fig. 3B). Levels of *SlS5H* expression were significantly lower in both mock and viroid infected transgenic lines, than the corresponding WT tomato plants. To find out if *RNAi_SlS5H* transgenic lines exhibited an activation of the defensive response against CEVd, the expression of the pathogenesis related protein 1 (*PR1*; accession X71592), which has been described as a classical marker of plant defence rapidly induced in CEVd-infected tomato plants [41, 42], was also studied by qRT-PCR at 2 wpi. As expected, *PR1* was induced by CEVd in WT plants, but also in both *RNAi_SlS5H* transgenic lines (Fig. 3C). Interestingly, levels of expression of *PR1* were already higher in mock-infected *SlS5H*-silenced tomato plants. These results appear to indicate that *SlS5H* silencing provokes the activation of the SA-mediated plant defence.

To better characterise the plant response of the *RNAi_SlS5H* transgenic lines upon CEVd infection, the expression of several defence genes was also studied at 3 wpi both in mock and infected plants. Regarding the gene silencing mechanisms that are activated by CEVd in WT plants, a significant reduction in *DCL1* and *DCL2* induction was observed in CEVd-infected *RNAi_SlS5H* lines when compared to the corresponding WT plants, thus indicating a correlation between the amount of CEVd and the induction of these two dicers (Fig. S4A and B). However, no significant differences were observed in the *RDR1* induction pattern in the transgenic plants, which displayed a significant induction of this gene upon viroid infection (Fig. S4C). As far as jasmonic acid (JA) response is concerned, a statistically significant reduction of the JA-induced proteinase inhibitor *TCI21* [43], was observed in both mock-inoculated *RNAi_SlS5H* lines when compared with WT (Fig. S4D), indicating that the final JA-mediated response is repressed in these tomato transgenic plants.

Therefore, we have observed that *RNAi_SlS5H* transgenic lines display constitutive *TCI21* repression as well as *PR1* overexpression, thus suggesting an increase of SA levels in these transgenic lines.

### Levels of SA and GA are altered in *RNAi_SlS5H* transgenic tomato plants upon CEVd infection

Free and total levels of SA and its hydroxylated product GA were quantified in the wild-type and *RNAi_SlS5H* transgenic lines upon viroid infection at 3 wpi (Fig. 3D and E). As expected, free and total SA and GA levels were higher in all the CEVd-infected plants.

In CEVd-inoculated plants, levels of total SA in both *RNAi_SlS5H* transgenic infected lines resulted to be significantly higher when compared with those observed in control infected plants, reaching 1000 nmol/g fresh weight whilst levels in control infected plants barely reached 400 nmol/g fresh weight (Fig. 3D). SA levels in non-pathogenic (mock) conditions were slightly higher in both transgenic lines, being statistically not significant when compared with wild-type plants.

Once detected the over-accumulation of SA, we studied the levels of GA, which is the product of the S5H activity. As Fig. 3E shows, a drastic reduction of total GA levels was observed upon viroid-infected in both *RNAi_SlS5H* transgenic lines as compared to the levels detected in the infected WT tomato plants (10-fold). Interestingly, this significant reduction was also observed in free GA corresponding to mock conditions. The presence of 2,3-DHBA was measured in all samples, and the levels were negligible. The higher levels of SA and the lower accumulation of GA found in the CEVd-infected *RNAi_SlS5H* transgenic lines confirm the decrease of the salicylate 5-hydroxylase activity in vivo and explain the observed enhanced resistance and activation of the SA-mediated plant defence in these transgenic plants.

### Infection with *Pseudomonas syringae* pv. *tomato* DC3000 produces specific phenolic accumulation pattern in* RNAi_SlS5H* transgenic tomato plants

**Fig. 4:**
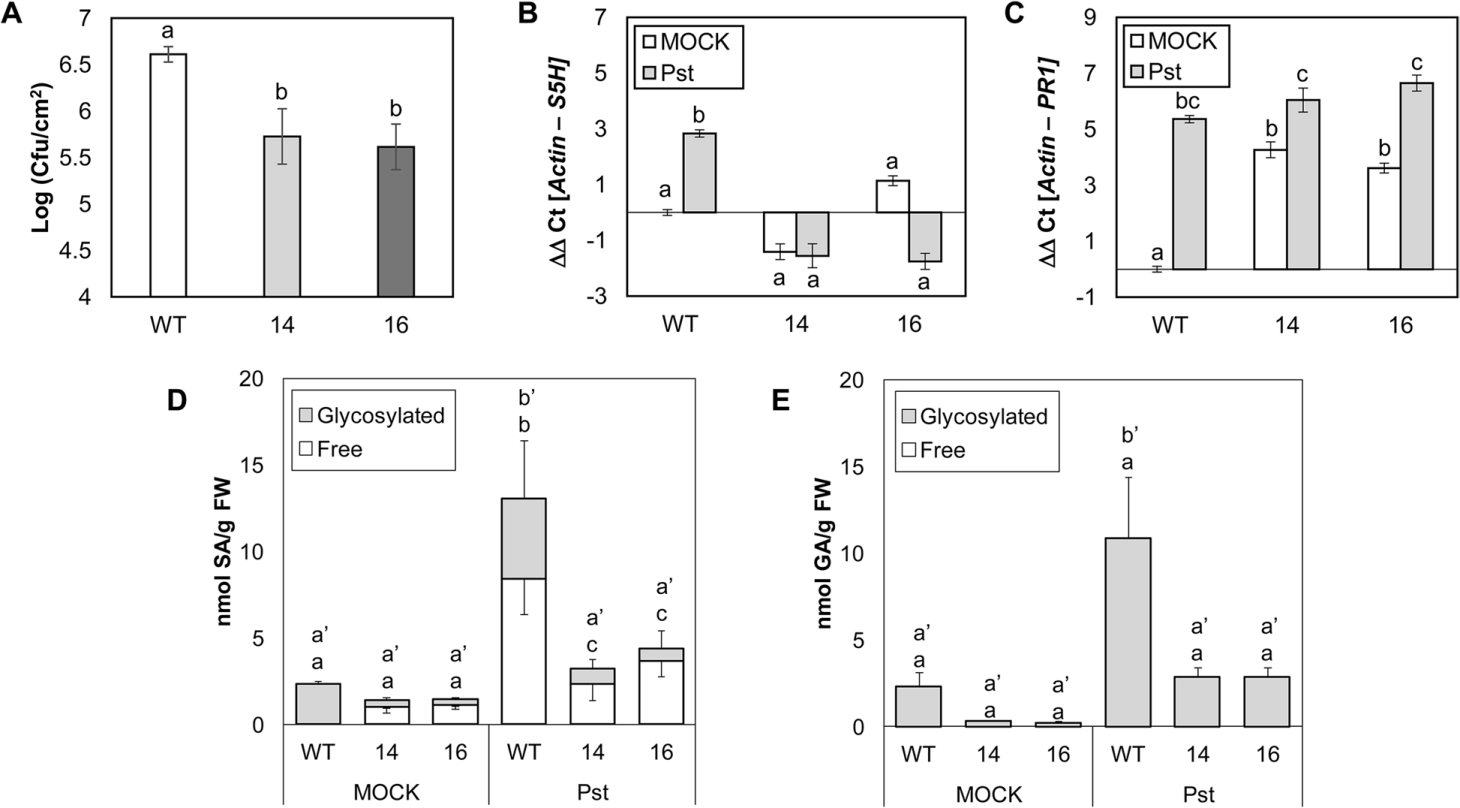
Characterization of wild type (WT) and transgenic *RNAi_SlS5H* (lines 14 and 16) tomato plants infected with *Pseudomonas syringae* pv. *tomato* DC3000 (*Pst*). Tomato plants were inoculated with *Pst* by immersion. Leaf samples were taken 24 hours after bacterial infection and analyzed for bacterial growth (**A**), *S5H *(**B**) and *PR1 *(**C**) gene expression, and accumulation of phenolic compounds (Salicylic Acid, SA, **D**; Gentisic Acid, GA, **E**). MOCK represents the non-inoculated plants. The qRT-PCR values were normalized with the level of expression of the actin gene. The data are presented as means ± standard deviation of a representative experiment (*n* > 3). Statistically significant differences (*p*-value < 0.05) between genotypes and infected or mock-treated plants are represented by different letters. Significant differences in SA and GA accumulation are analysed in free (x) and total (x’) phenolic compounds

Bacterial infection of tomato plants produces lower accumulation of SA when compared with levels produced by CEVd infection [37]. To study the role of *SlS5H* in this tomato-pathogen interaction, WT and *RNAi_SlS5H* transgenic tomato plants were infected with the virulent strain of *Pst*, and bacterial counting from infected leaves was carried out at 24 h after infection (hpi). As shown in Fig. 4 A, a significant 10-fold decrease in the number of colony forming units (CFUs) was observed in infected tissues of the different transgenic lines with respect to their genetic background, confirming that transgenic plants *RNAi_SlS5H* also exhibited resistance against *Pst*.

*SlS5H* silencing in tomato plants upon bacterial infection was also confirmed by qRT-PCR, being differences in *SlS5H* expression in bacterial infected *RNAi_SlS5H* transgenic plants statistically significant compared to the expression levels observed in the infected WT (Fig. 4B). Similar to what was observed upon viroid infection; *PR1* expression was already higher in mock-inoculated *SlS5H*-silenced tomato plants and was significantly induced by bacteria in both WT and *RNAi_SlS5H* transgenic lines (Fig. 4C).

In a similar manner to that performed with CEVd-infected plants, SA and GA levels were measured in both WT and *RNAi_SlS5H* transgenic plants upon bacterial infection (Fig. 4D and E). In *Pst*-tomato interaction, the levels of free and total SA were induced by the hemibiotrophic pathogen in all the analysed plants. As previously observed in mock-inoculated plants for CEVd infection, no statistically significant differences were observed in mock-inoculated transgenic plants, when compared with WT. Nevertheless, the slight overaccumulation of free SA in both mock-inoculated transgenic plants could explain the PR1 induction observed in these plants (Fig. 4C). Strikingly unlike what was observed upon CEVd infection, the bacteria produced a significant reduction of free and total SA levels in both *RNAi_SlS5H* transgenic lines when compared with levels observed in *Pst*-infected WT plants (Fig. 4D). Regarding GA, only WT tomato plants showed a statistical accumulation of total GA levels after bacterial infection, thus indicating that the product of *SlS5H* is also reduced in *RNAi_SlS5H* transgenic plants upon bacterial infection (Fig. 4E).

The reduction of SA levels observed in *RNAi_SlS5H* transgenic lines upon bacterial infection indicates that SA undergoes a specific catabolic process upon pathogen infection in these transgenic plants.

### A metabolomic analysis of the *RNAi_SlS5H* transgenic tomato plants upon viroid and bacterial infection reveals differences in SA metabolism upon pathogen attack

**Fig. 5:**
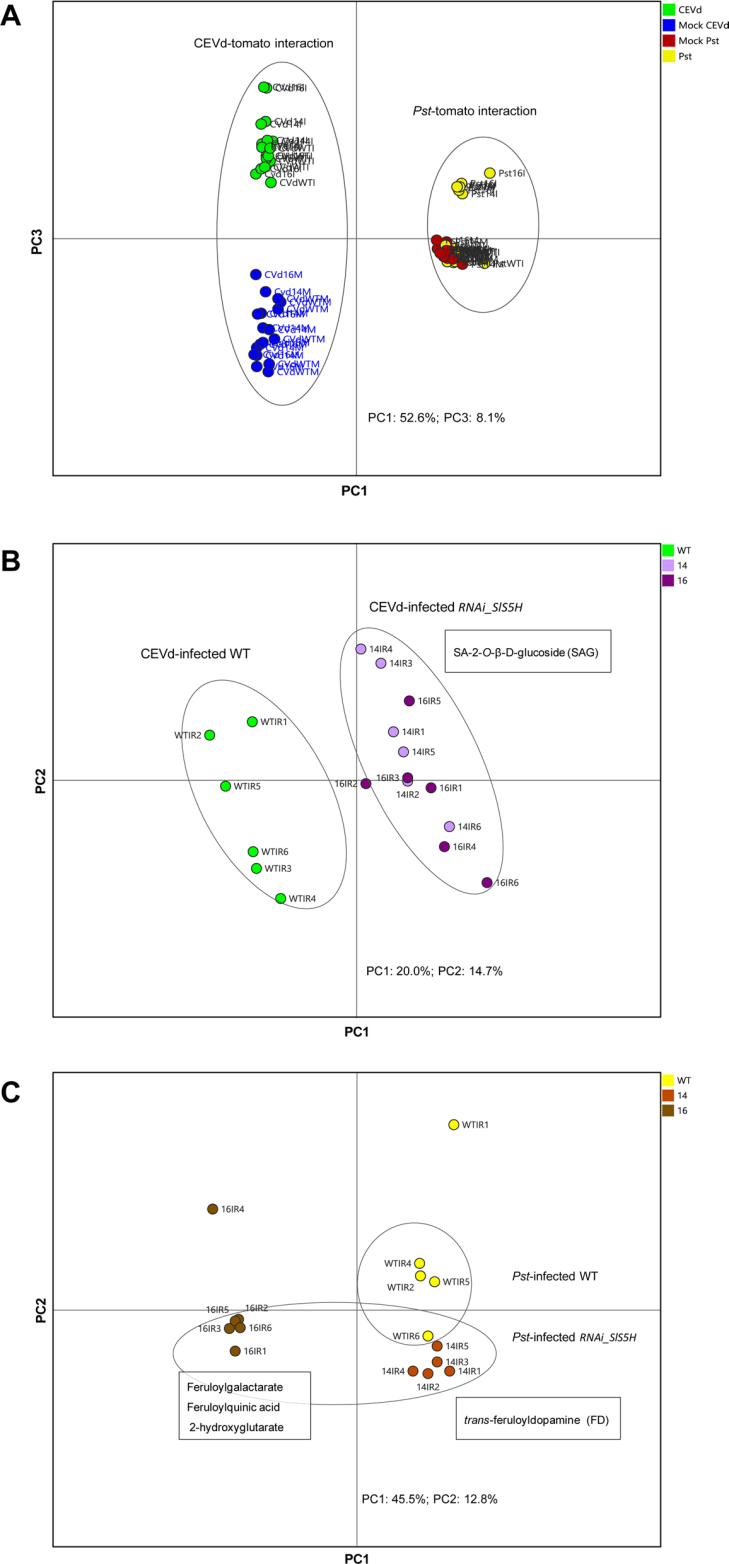
PCA Score plots based on whole range of on the whole array of the mass spectra within a *m/z* range from 100 to 1500 using unit variance (UV) scaling method of methanolic extracts from tomato leaves. **A** Green: Infected by CEVd (3 wpi); Blue: Mock for CEVd infection; Yellow: Infected by *Pst* (24 hpi); Red: Mock for bacterial infection. **B** Green: Wild type (WT); light purple: CEVd infected *RNAi_SlS5H* 14 at 3 wpi; dark purple: CEVd infected *RNAi_SlS5H* 16 at 3 wpi. **C** Yellow: Wild type (WT); orange: *Pst* infected *RNAi_SlS5H* 14 at 24 hpi; brown: *Pst* infected *RNAi_SlS5H* 16 at 24 hpi

To better understand the SA metabolism in *RNAi_SlS5H* transgenic lines upon each infection, a metabolomic study based on ultra-performance liquid chromatography-mass spectrometry (UPLC-MS) was performed. For viroid infection, 8-day-old tomato plants were used, and samples were collected 3 weeks after CEVd inoculation (wpi), while bacterial infection was carried out on 5-week-old tomato plants and the harvesting time was 24 h after *Pst* infiltration (hpi). Then, hydroalcoholic extracts from control and infected *RNAi_SlS5H* tomato leaves were analysed by UPLC-MS, and multivariate data analysis was employed to deal with the large number of mass data. Specifically, a principal component analysis (PCA) was first applied to identify metabolic changes after viroid and bacterial infection of tomato plants (Fig. 5A). An extensive separation between both tomato interactions was observed by PC1 due to the different experimental conditions: temperature and plant age. Reaching the PC3, the metabolic changes in tomato leaves produced by both pathogens were explained, being greater those caused by viroid inoculation. To exclude the differences due to this biological variability, two different PCA were therefore applied separately on each tomato-interaction.

In particular, the first two components of the PCA score plot of viroid-tomato interaction (Fig. S5A) divided the observations by the infection (mock *vs.* infected plants; PC1) and genotype (WT *vs.* transgenic plants; PC2). In order to elucidate the SA metabolism in tomato plants against CEVd, a PCA of both infected WT and transgenic *RNAi_SlS5H* plants was performed (Fig. 5B). For the identification of the metabolites accumulated in the infected *SlS5H* silenced lines (14 and 16), the positive PC1 loading plot was analysed. Interestingly, the glycosylated form of SA (SAG) was the most accumulated compound in the transgenic plants (fold change transgenic lines *vs.* WT: 3.0; *p*-value 0,009). These results are in accordance with the total SA accumulation measured by HPLC-fluorescence in CEVd infected *RNAi_SlS5H* plants (Fig. 3D).

In the case of bacteria-tomato interaction, the PC3 of PCA (Fig. S5B) explained the different metabolic content of transgenic tomato plants from WT, while PC1 clearly discriminated the metabolome of the infected *RNAi_SlS5H* line 16. Similarly to CEVd interaction, the PCA of infected tomato plants was required to investigate the role of SA in the bacterial infected tomato plants (Fig. 5C). By analysing the negative PC1 and PC2 loading plot, the metabolites accumulated in both transgenic lines were identified. In contrast to CEVd infection, SAG accumulation was not induced by *Pst* in both transgenic lines according to the results obtained using fluorescence-based detection (Fig. 4D). In tomato-bacteria interaction, some of the compounds over-accumulated in the transgenic infected leaves were identified as feruloyldopamine (fold change between transgenic lines and WT: 5.3; *p*-value 0.003), feruloylquinic acid (fold change: 5.1; *p*-value 0.006), feruloylgalactarate (fold change: 3.4; *p*-value 0.01) and 2-hydroxyglutarate (fold change: 1.4; *p*-value 0.04).

### *SlS5H *silencing reveals differences in SA biosynthesis gene expression upon pathogen attack

**Fig. 6:**
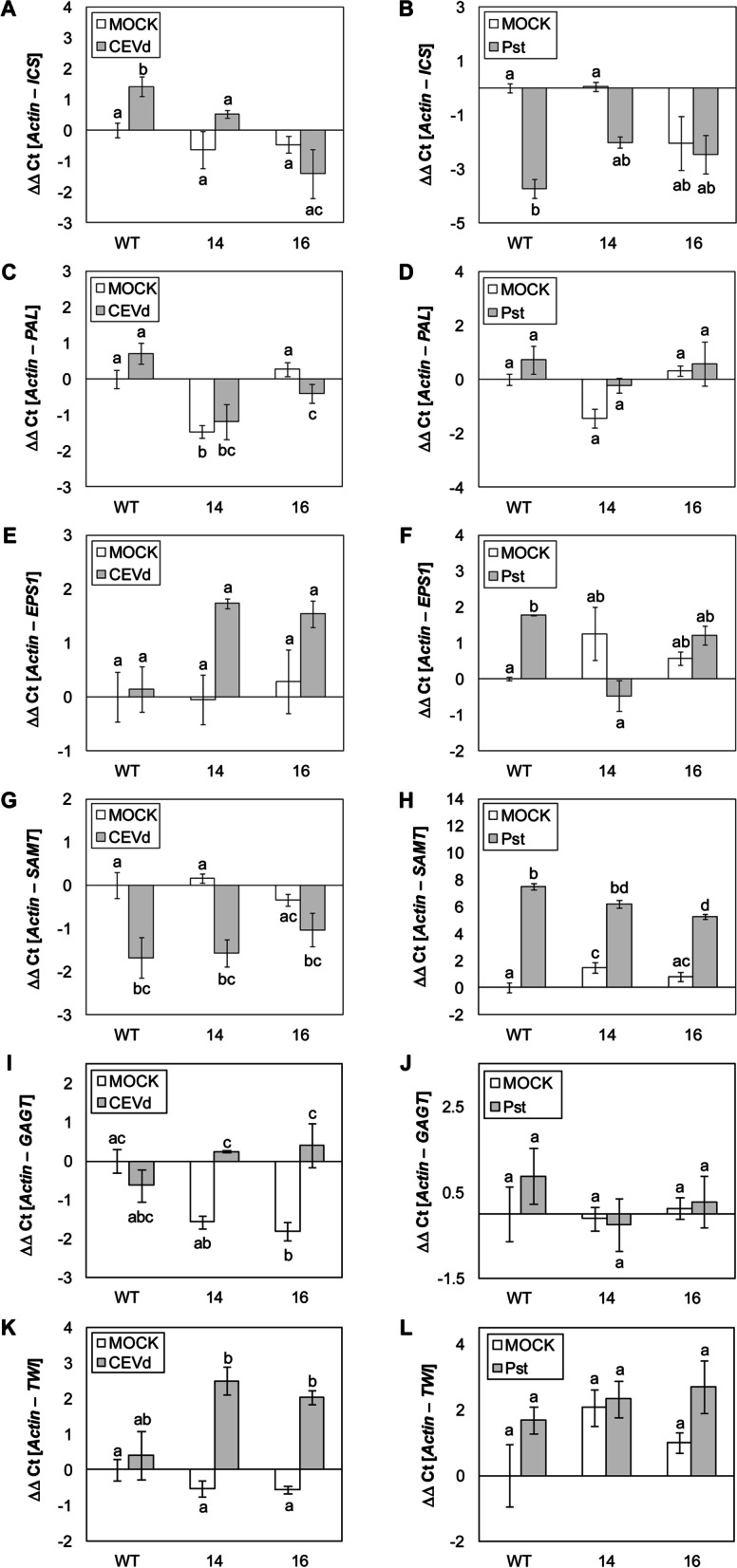
Differences in the pattern of induction of SA metabolism genes upon viroidal (CEVd) and bacterial (Pst) inoculation in wild type (WT) and *RNAi_SlS5H* (lines 14 and 16) transgenic tomato plants by qRT-PCR. *ICS* (**A**), *PAL* (**C**), *EPS1* (**E**), *SAMT* (**G**), *GAGT* (**I**) and *TWI* (**K**) tomato gene expression levels 3 weeks post-inoculation with CEVd; *ICS* (**B**), *PAL* (**D**), *EPS1* (**F**), *SAMT* (**H**), *GAGT* (**J**) and *TWI* (**L**) gene expression levels 24 h after inoculation with the bacteria *Pseudomonas syringae* DC3000; MOCK represents the mock-inoculated plants. The qRT-PCR values were normalized with the level of expression of the actin gene. The expression levels correspond to the mean ± the standard error of a representative experiment (*n* = 3). Statistically significant differences (*p*-value < 0.05) between genotypes and infected or mock-inoculated plants are represented by different letters

To study differential expression of genes participating in SA biosynthesis and how they are affected by silencing *SlS5H*, qRT-PCR were performed for *ICS* (Solyc06g071030 or XM_019214145), *PAL* (Solyc09g007890 or NM_001320040), *EPS1* (Solyc08g005890 or XP_004244447), *SAMT* [44], and the glycosyltransferases of phenolic compounds *GAGT* [31] and *Twi1* [45]in samples corresponding to CEVd or *Pst* infections for both WT and *RNAi_SlS5H* transgenic plants (Fig. 6).

As Fig. 6 A shows, a significant induction in *ICS* was observed in WT plants upon CEVd infection, being that induction impaired in the transgenic lines, thus suggesting that the SA biosynthesis is down-regulated when the SA hydroxylation is prevented, which may lead to SA over-accumulation. Contrasting with CEVd infection, a clear reduction of *ICS* expression was detected upon bacterial infection, in both WT and transgenic lines. However, this decrease of expression was not statistically significant in the transgenic plants (Fig. 6B).

As far as *PAL* pattern of expression is concerned, whilst CEVd infection caused a significant reduction in both transgenic lines compared to infected WT, no significant differences caused by *Pst* infection were observed (Fig. 6 C and D).

The last step in the SA biosynthesis through the isochorismate pathway involves the conversion of isochorismate-9-glutamate into SA, being performed by *EPS1*. Whilst WT plants appear not to display any significant induction of *EPS1* by CEVd, bacterial infection clearly provoked the induction of this gene. This pattern was completely opposite in both *RNAi_SlS5H* transgenic lines, since they showed a slight induction of *EPS1* upon CEVd infection, being impaired in the expression of *EPS1* upon bacterial infection (Fig. 6E, F).

Moreover, a reduction of *SAMT* expression upon CEVd infection was measured in all the genotypes, with no significant differences observed between WT and transgenic plants. In contrast, *SAMT* induction caused by bacterial infection was lower in transgenic plants, showing statistically significant differences between the induction observed in infected WT and *RNAi_SlS5H 16* transgenic plants (Fig. 6H).

Finally, a far as the glycosyltransferases of phenolic compounds *GAGT* and *Twi1* are concerned, whilst no statistical differences were observed in the transgenic plants infected with *Pst*, a slight increase in the induction of both glycosyltransferases was observed upon viroid infection (Fig. 6I-L).

Although the differences observed in both viroid and bacterial infections were not statistically significant for all the analysed genes, a noticeable variation in the pattern of expression of several genes participating in SA metabolism was detected upon infection by these two pathogens, thus suggesting that SA homeostasis has specific differences for each tomato-pathogen interaction.

### *SlS5H* silencing causes repression of the JA defence response

**Fig. 7:**
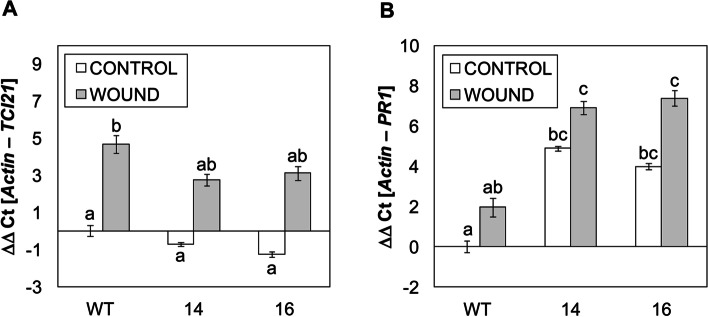
Expression of the tomato *TCI21* (**A**) and *PR1* (**B**) genes in wild type plants (WT) and the *RNAi_SlS5H* (lines 14 and 16) transgenic tomato plants in control (CONTROL) and wounded (WOUND) plants. Samples were taken 24 h after wounding; CONTROL represents the non-wounded plants. The qRT-PCR values were normalized with the level of expression of the actin gene. The expression levels correspond to the mean ± the standard error of a representative experiment (*n* = 3). Statistically significant differences (*p-*value < 0.05) between genotypes and infected or mock-inoculated plants are represented by different letters

Once confirmed that *SlS5H* silencing provokes an activation of the SA-mediated defence, we studied the possible cross-talk with the JA-mediated response. To that purpose, control and *RNAi_SlS5H* transgenic lines were wounded, and the induction of *TCI21* was studied at 24 h after wounding (Fig. 7A). As expected, *TCI21* was highly induced by wounding in WT plants, whilst *RNAi_SlS5H* wounded plants displayed only a slight induction of *TCI21*, which was comparable to non-wounded plants, thus indicating that JA response is repressed in these transgenic plants. Interestingly, a higher induction of *PR1* was detected in the *SlS5H*-silenced plants both in non-wounded and upon wounding when compared with the WT plants (Fig. 7B), reinforcing the observed activation of SA-mediated defence in the *RNAi_SlS5H* transgenic plants.

**Fig. 8:**
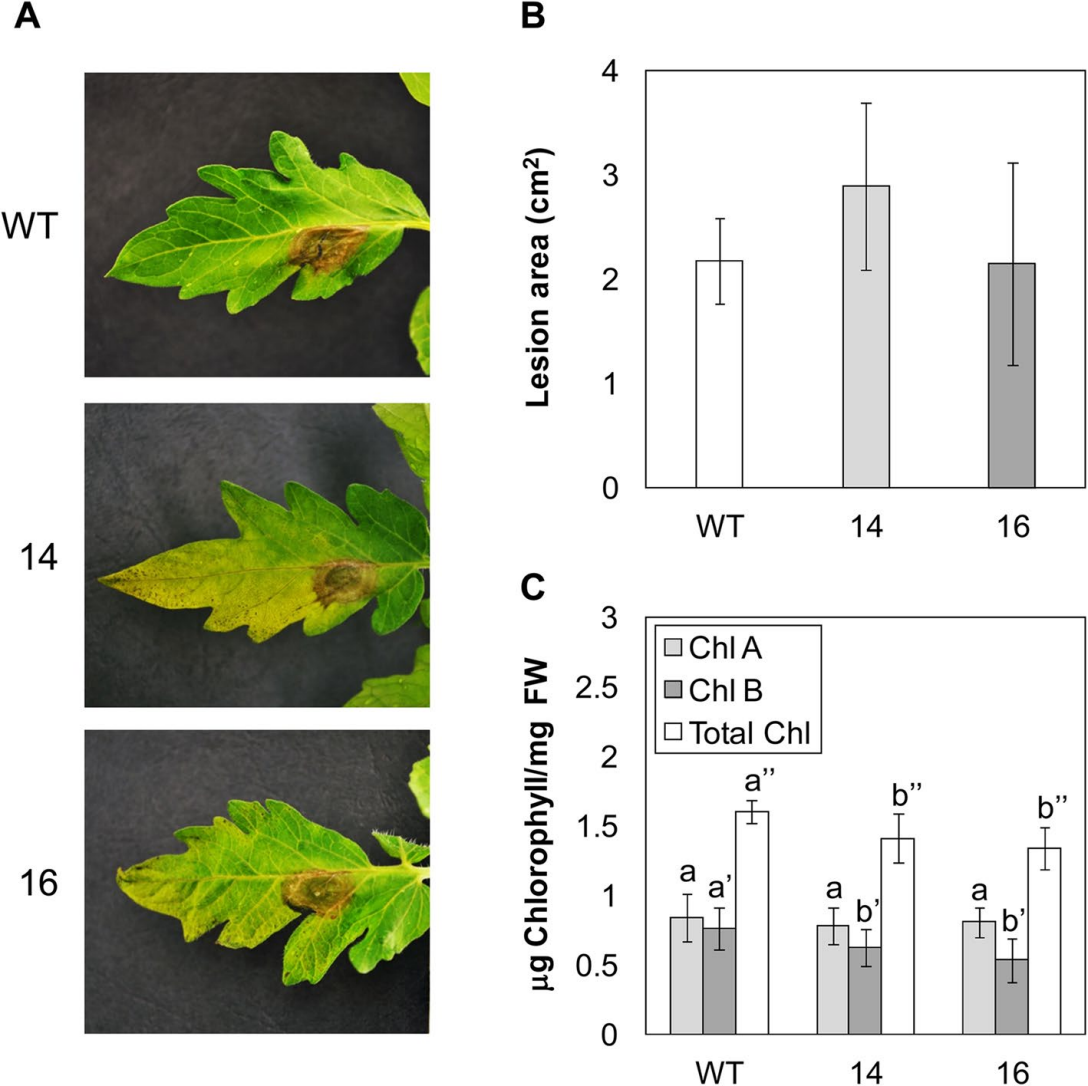
Response of wild type plants (WT) and *RNAi*_S5H transgenic lines 14 and 16 to *Botrytis cinerea* infection. Representative images (**A**), lesion area (**B**) and chlorophyll content (**C**) of plants infected with *B. cinerea* 5 days after fungal inoculation. Bars represent the mean ± the standard deviation of a representative experiment (*n* = 6). Significant differences between genotypes are represented different by letters since *p-*value < 0.05

Since JA response appeared to be repressed in *RNAi_SlS5H* transgenic lines, the phenotype against the necrotrophic fungal pathogen *Botrytis cinerea* was explored (Fig. 8A). Although no statistically significant differences were found in the size of necrotic lesions between the transgenic lines and the corresponding parental plants (Fig. 8B), both *RNAi_SlS5H* transgenic lines displayed an increase in the susceptibility against *Botrytis cinerea*, as suggested by the increase in the yellowish area shown by the transgenic plants. To better quantify this effect, chlorophyll content was measured in control and transgenic plants infected with the fungus. As Fig. 8C shows, *RNAi_SlS5H* transgenic lines accumulated significant lower levels of chlorophyll B and total chlorophylls, therefore confirming the observed increased susceptibility of *RNAi_SlS5H* transgenic lines against *Botrytis cinerea.*

### Silencing of *SlS5H* results in early senescence

Previous research on salicylate hydroxylase activity in Arabidopsis reported that the deficiency in this enzyme provokes an advanced senescent response [21, 23]. However, no noticeable phenotypic differences have been reported in *SlDMR6-1* tomato mutants [24].

**Fig. 9:**
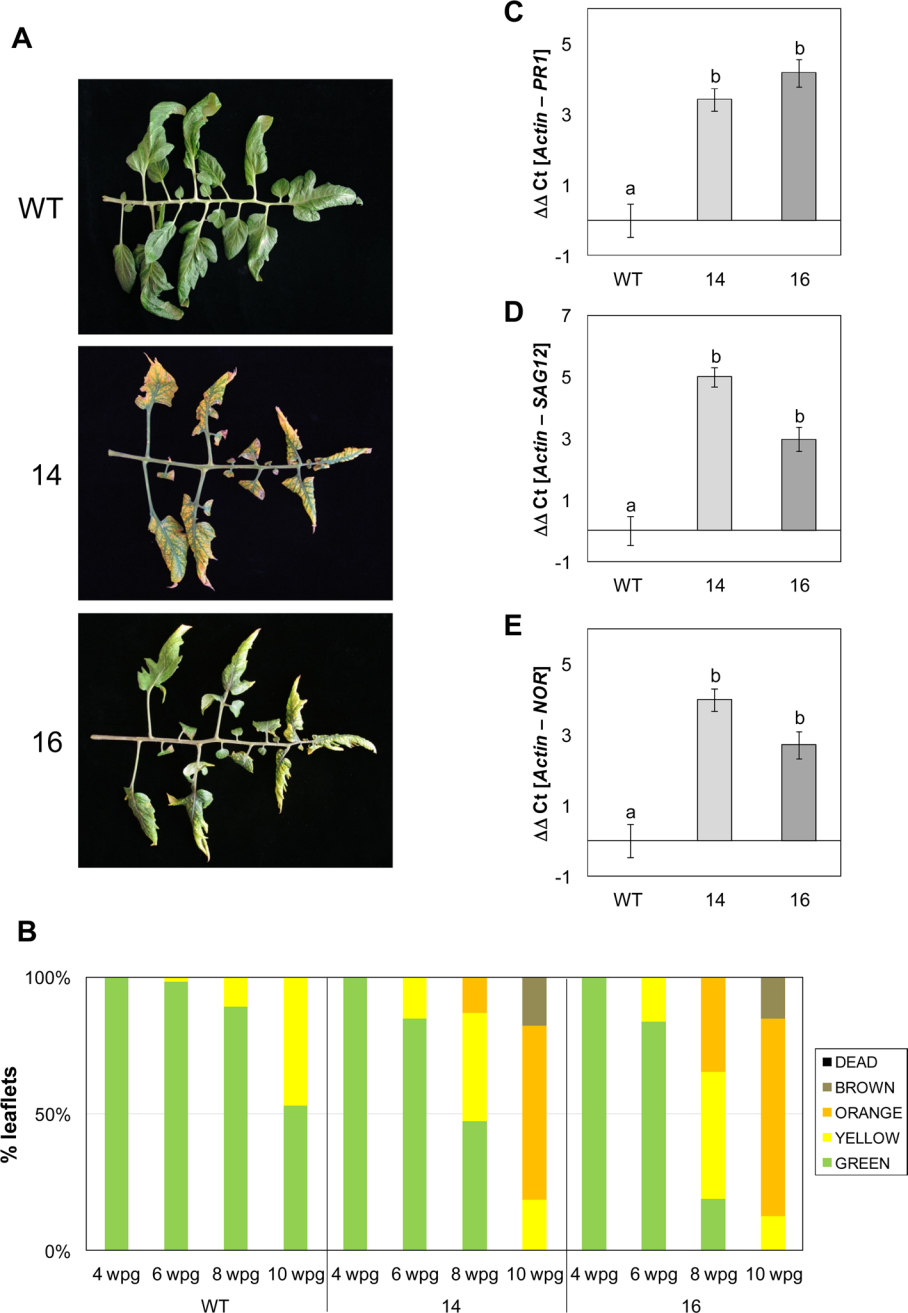
Early senescent phenotype and senescence-related gene expression analysis in WT and *RNAi*_S5H transgenic lines 14 and 16. **A** Representative images and **B** evolution of senescence in WT and *RNAi_S5H* tomato leaflets. The percentage of leaflets of same appearance with respect to the total of leaflets corresponding to the third, fourth and fifth leaves of the WT and *RNAi_SlS5H* transgenic tomato plants at 4, 6, 8 and 10 weeks post-germination (wpg) is shown. “GREEN” refers to the natural color of the tomato leaves, “YELLOW” when the leaves begin to age and acquire yellowish spots, “ORANGE” is the time when the leaf turns completely yellow, “BROWN” when necrotic lesions appear due to aging and “DEAD” defines those leaves that have detached from the plant or that are completely necrotic. Expression levels of *PR1 *(**C**), *SAG12 *(**D**) and *NOR *(**E**) are shown for WT plants and *RNAi_SlS5H* transgenic lines 14 and 16, 4 weeks after germination. The qRT-PCR values were normalized with the level of expression of the actin gene. The expression levels correspond to the mean ± the standard error of a representative experiment (*n* = 3). Statistically significant differences (*p-*value < 0.05) between genotypes are represented by different letters

To study the developmental phenotype of *RNAi_SlS5H* transgenic tomato plants, 5 individuals for each genotype were grown for 10 weeks, and the percentages of leaflets displaying different senescence stages were recorded. As Fig. 9 A shows, *RNAi_SlS5H* transgenic plants displayed and earlier chlorosis, even leading to the leaf collapse. Particularly, in 10-week-old plants we observed that control plants still possess approximately 50% of the green leaves, while hardly any leaf of the transgenic lines remained green, turning the entire observed leaves to different intensities of yellow and brown, eventually leading to leaf fall (Fig. 9B).

To better characterize the phenotype of the *RNAi_SlS5H* transgenic tomato plants, we measured weight and conductivity at 10 weeks after germination, observing a significant weight reduction in the transgenic plants (Fig. S6A). Moreover, electrolyte leakage, which is a hallmark of cell death, was also significantly increased in both transgenic lines silencing *SlS5H* (Fig. S6C). Finally, the chlorophyll content was also measured, observing significant lower levels of chlorophyll B and total chlorophylls in *RNAi_SlS5H* transgenic tomato leaves (Fig. S6D).

To reinforce the data obtained by phenotypic visualization, a gene expression analysis for *PR1* as well as the senescence markers *SAG12* (AT5G45890 tomato ortholog; Solyc02g076910) and *NOR* [46] was carried out by qRT-PCR at 4 weeks after germination. As shown in Fig. 9C, a significant increase of *PR1* expression was observed in *RNAi_SlS5H* transgenic lines with respect to control plants, which correlates with the higher SA levels previously observed in mock-inoculated plants (Fig. 4D). In parallel with *PR1* expression levels, both senescence markers *SAG12* (Fig. 9D) and *NOR* (Fig. 9E) were differentially upregulated in the *RNAi_SlS5H* transgenic leaves, confirming the developmental phenotype of early senescence observed in these plants.

**Fig. 10:**
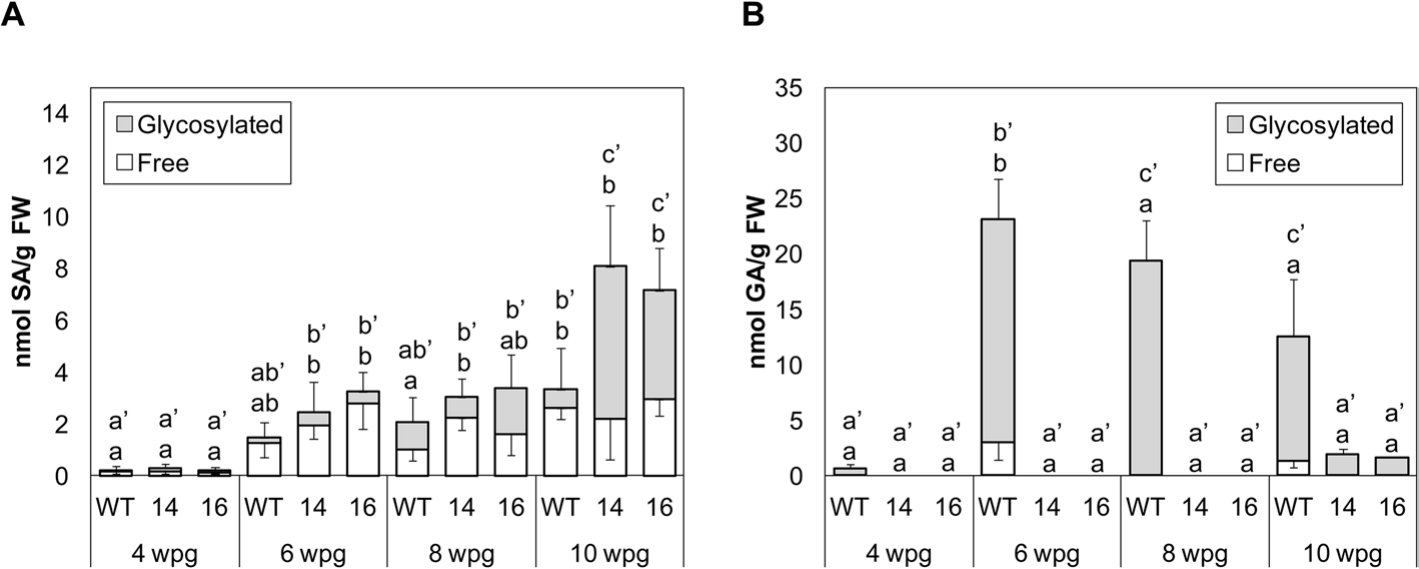
Evolution of free and glycosylated SA (**A**) and GA (**B**) accumulation in wild type (WT) and *RNAi_SlS5H* (lines 14 and 16) transgenic tomato plants. Samples were taken 4, 6, 8 and 10 weeks post-germination (wpg). Leaf extracts were analyzed by fluorescence HPLC. The data are presented as the mean ± standard deviation of a representative experiment (*n* = 6). Significant differences for free and glycosylated SA and GA accumulation between different genotypes are represented by different letters since *p-*value < 0.05

Levels of free and total SA and GA were measured at different stages of development (Fig. 10). To that purpose, samples were collected at the indicated time points and free and total levels of both phenolics were measured by HPLC in both control and *RNAi_SlS5H* transgenic tomato plants. In general, levels of SA were higher in the transgenic plants impaired in 5-hydroxylation, being differences in total SA with control plants statistically significant at 10 weeks after germination. However, levels of GA were found to be lower at every time points, being total GA levels statistically significant from 6 weeks after germination, which confirms that the reduction of GA in these transgenic plants is consistent and reproducible in all the analysed samples.

Therefore, our results appear to indicate that over-accumulation of SA in the *RNAi_SlS5H* transgenic tomato plants provokes toxicity, which may explain their lower growth rates and early senescence.

## Discussion

Resistance to pathogens is associated with SA-mediated activation of plant immune response, being SA a key defence phytohormone with many potential derived applications in agriculture [6]. The fine-tuned regulation of SA accumulation constitutes an important point in plant immunity [47, 48]. This regulation can occur at different levels, including the control of its biosynthesis as well as its catabolism. Hydroxylation of SA to form 2,3-DHBA and GA can be considered part of its catabolism, but these forms also constitute a temporary source for SA, since their production could be reversible. Here we study the effect of the impairment of SA hydroxylation in tomato plants subjected to CEVd and *Pst* infections, which produce high and low levels of GA, respectively [33, 36–38]. Collectively, our findings provide insight into the modulation of SA metabolism upon biotic stress and provide new insights to previous results on this topic [9, 47].

The SA-5 hydroxylation is performed by *AtS5H/DMR6* in *Arabidopsis thaliana*, displaying *s5h* mutants over accumulation of SA and enhanced resistance to *Pst* [23]. We have identified the tomato *S5H* ortholog and studied its induction upon different pathogen infections which provoke SA and GA accumulation, including CEVd, TSWV, ToMV and a virulent and an avirulent strain of *Pst* (Fig. 1). Similar results have been reported by Thomazella et al. [24] from publicly available transcriptome data, including the bacteria *Xanthomonas gardneri* and *Pst*, the oomycete *Phytophthora capsici*, and the fungus *Moniliophthora perniciosa* as pathogens that trigger *SlS5H-DMR6-1* induction. In general terms our data suggest a correlation between *SlS5H* induction and symptomatology. In accordance, its induction correlates not only with symptom development but also with SA and GA accumulation in tomato, since both phenolics have been described to accumulate at very high levels in tomato plants infected by CEVd [33, 36–38] which provokes a high induction of *SlS5H* itself. According to that, *SlS5H* was also found to be induced by its own substrate at 6 hpt (Fig. S2A), thus indicating a SA-mediated regulation of *SlS5H* expression. However, the higher induction of *SlS5H* at 24 h upon avirulent bacterial infection (Fig. 1B) appear to indicate that the role of this gene is more than detoxifying elevated levels of SA generated. Whether that regulation is direct or may occur through the participation of different molecular elements remains unclear. Our results reinforce the role of *SlS5H*, also known as *SlDMR6-1*, in SA metabolism.

SlDMR6-1 was described to be a 2-oxoglutarate-Fe(II) oxygenase acting on SA to produce GA in vitro [24]. Here we confirm its in vivo SA 5-hydroxylase activity, by transiently overexpressing *SlS5H* in *Nicotiana benthamiana*. Plants agro-inoculated with a *35S:SlS5H* construction and further embedded with SA displayed lower levels of free and total SA, confirming that this phytohormone is a substrate for SlS5H in vivo, although a GA over-production was not detected (Fig. S3). These results agree with those previously described in SA-treated *Nicotiana tabacum* plants which did not display GA accumulation [33], probably indicating than this GA is rapidly catabolised in SA-embedded plants belonging to the *Nicotiana* genus. Our results confirm the extraordinary versatility of SA metabolism in different plant species, differences being reported in SA and GA accumulation of up to 100-fold [23].

Given the quantitative importance of GA accumulation in CEVd-infected tomato plants [33, 36–38], we decided to study the role of *SlS5H* in this interaction by analysing the phenotype of *RNAi_SlS5H* transgenic tomato plants upon viroid infection. *SlS5H* silencing resulted in a very low GA accumulation, and produced an enhanced resistance to CEVd, reducing both symptoms and viroid accumulation (Figs. 2 and 3 A). Our results further extend the described broad spectrum resistance mediated by inhibition of SA hydroxylation [24] to a non-coding, RNA pathogen.

This enhanced resistance observed in *SlS5H*-silenced plants was accompanied by an induction of the SA-mediated plant defence response, since *PR1* expression was higher in viroid infected *RNAi_SlS5H* transgenic tomato plants when compared to the corresponding infected non-transgenic plants (Fig. 3C). In contrast, activation of the silencing mechanisms through *DCL1* and *DCL2* was reduced in *RNAi_SlS5H* transgenic tomato plants (Fig. S4A and B), thus indicating these mechanisms are related to the viroid progression, which is limited in the transgenic plants, but not to the SA accumulation itself. In contrast, a correlation between SA accumulation and activation of silencing mechanisms has been previously described [35], thus suggesting a role of SA in plant silencing. Besides, the JA-mediated response appeared to be down-regulated in the *RNAi_SlS5H* transgenic tomato plants, displaying a lower induction of *TCI21* upon CEVd infection and wounding (Figs. S4D and 7A), and showing higher susceptibility to *Botrytis cinerea* (Fig. 8). The reciprocal antagonism between JA and SA [49] and the higher induction of *PR1* in the transgenic lines points to a SA overaccumulation, which could be responsible for the observed SA-mediated plant resistance to CEVd. According to this, *RNAi_SlS5H* transgenic tomato plants displayed higher levels of total SA and significantly lower levels of total and free GA, confirming the decrease of SlS5H activity *in vivo.* The enhanced resistance of *Sldmr6-1* lines to different classes of pathogens, such as bacteria, oomycetes and fungi has also been correlated with increased SA levels and transcriptional activation of immune responses [24].

Tomato plants infected with *Pst* have been described to display a different pattern of accumulation of both SA and GA when compared to CEVd infected tomato plants, reaching SA levels less than 10 nmol/g, and around 40 nmol/g for GA [37], which are much lower than those detected upon CEVd infection [33, 36–38]. To test the effect of *SlS5H* impairment on the tomato response to bacteria, similar studies to those performed for CEVd were carried out. Our results were in accordance with the enhanced *PR1* induction (Fig. 4C), enhanced resistance (Fig. 4A), and reduced GA accumulation in *RNAi_SlS5H* transgenic tomato plants triggered by bacteria (Fig. 4E). Unlike CEVd infection, an increase in SA accumulation was not detected in transgenic tomato plants upon *Pst* infection, thus indicating that the catabolism of SA shows particularities that are dependent on the plant-pathogen interaction (Fig. 4D).

The availability of different SA-deficient mutants and *NahG* transgenic plants in *Arabidopsis thaliana* background has provided vast information on the SA metabolism in response to bacterial infection in this species [7, 12]. However, fewer studies have been done on the role of SA in tomato plants in response to bacteria. Therefore, to better understand the modulation of SA metabolism by *Pst* infection in tomato, and to compare it with that activated by CEVd, metabolomic analyses were performed using our *SlS5H*-silenced plants as a tool to study the effect of the altered levels of SA. Our metabolomic assay confirmed that the glycosylated form of SA was the most accumulated compound in the *RNAi_SlS5H* transgenic tomato upon viroid infection (Fig. 5B), whereas the glycosylated GA is described to be the most accumulated compound in CEVd-infected WT plants [38], thus confirming in vivo the proposed SA 5-hydroxylase activity for SlS5H. Strikingly, the impairment of SA 5-hydroxylation in tomato plants uncovered alternative SA homeostasis routes upon *Pst* infection, since *RNAi_SlS5H* transgenic plants over-accumulated feruloyldopamine, feruloylquinic acid, feruloylgalactarate and 2-hydroxyglutarate (Fig. 5C).

Feruloyldopamine is a hydroxycinnamic acid amide (HCAA) known to accumulate in tomato plants infected with a high *Pst* bacterial titre, thus producing a hypersensitive-like response [50, 51]. It is worthy to note that this HCAA accumulation was accompanied by a rapid and sharp production of SA. These results appear to indicate that SA over-accumulation produced by bacterial infection could trigger the accumulation of this HCAA to prevent a toxic SA effect. The acyl-quinic acids are a diverse group of plant-derived compounds produced principally through esterification of an hydroxycinnamic acid and quinic acid [52]. Particularly, the flavonoid feruloylquinic acid has been described to accumulate in barley leaves upon ultraviolet and photosynthetically active radiation suggesting a protective role [53]. In *Coffea canephora*, feruloylquinic acid is accumulated in juvenile leaves associated with chloroplasts, therefore suggesting a protective role against photooxidative damage [54]. Feruloylgalactarate results from the reaction of feruloyl-CoA with galactaric acid. The accumulation of hydroxycinnamic acids esters of quinic acid and glucaric acids has also been described in tomato plants infected with *Ralstonia solanacearum*, the causal agent of bacterial wilt, a highly destructive bacterial disease [55]. Finally, the accumulation of 2-hydroxyglutarate has been proposed to be linked to light-dependent photorespiration, it is related to oxidative stress and is considered as a marker for senescence in plants [56, 57]. In this respect, the expression of 2-hydroxyglutarate dehydrogenase increases gradually during developmental and dark-induced senescence in *Arabidopsis thaliana* [58].

The accumulation of all these compounds may be somehow related with photooxidative damages, caused by biotic and abiotic stresses or senescence. There is a well-known interplay between SA and reactive oxygen species (ROS), being ROS signals involved both upstream and downstream SA signalling [59]. Therefore, the observed over-accumulation of these metabolites in the *RNAi_SlS5H* plants upon bacterial infection could be exerting a protective role against the photooxidative damage provoked by the transient over-accumulation of SA which could be promoting ROS production, as previously described [59]. On the other hand, there is a biosynthetic connection between SA and these phenolic compounds, displaying age-related differences in their biosynthesis in *Solanum lycopersicum* cv. *amateur* infected by *Pst* [60]. Therefore, the accumulation of feruloyldopamine, feruloylquinic acid and feruloylgalactarate could also be the result of the SA catabolism.

The specific pathogen-triggered SA metabolism observed in this study, was accompanied with noticeable differences in the pattern of induction of SA biosynthesis genes provoked by the CEVd and *Pst* (Fig. 6). Regarding *ICS* and *EPS1* expression levels, a significant induction was observed in WT plants after viroid and bacterial infection, respectively. However, no differences were observed after CEVd and *Pst* infection in transgenic plants when compared with mock controls. *ICS* encodes an isochorismate synthase in the plastids, being required for SA biosynthesis [7]. The impairment of its induction in transgenic plants upon CEVd infection appears to indicate a negative feed-back produced by SA over-accumulation (Fig. 6A). However, this negative feed-back was not detected neither in transgenic plants upon bacterial infection, where the levels of SA were lower than after a viroid infection (Fig. 7A), nor at *EPS1* level, which is described to act at the cytoplasm [12]. *EPS1* was significantly induced by *Pst* in WT tomato plants, while *RNAi_SlS5H* plants did not display that induction. In the same way, a reduction of the *PAL* expression was observed in transgenic plants upon CEVd infection coinciding with SA over-accumulation, and no differences in *PAL* induction were detected in tomato-*Pst* interaction. Regarding WT tomato plants, *PAL* was not induced by any of the pathogens studied here, indicating that the main source of SA upon infection is also dependent on the IC pathway in tomato plants, as previously described in Arabidopsis [8, 9].

Finally, the *SAMT* regulation was opposite depending on the attack of the different studied pathogens in WT plants. While viroid caused a significant *SAMT* repression, bacterial infection produced *SAMT* induction. The reduced expression of *SAMT* upon CEVd inoculation could be due to the lower amount of its substrate which is redirected to form SAG (Figs. 3D and 5B), since a correlation between SA and MeSA accumulations has been previously described [61]. However, the *SAMT* induction in tomato plants upon bacterial infection matches with the increase in the production of methyl salicylate (MeSA), salicylaldehyde, and ethyl salicylate described in this virulent plant-pathogen interaction [37]. The lower induction of *SAMT* upon *Pst* infection in transgenic plants could also be due to the lower amount of its substrate. Together, these results confirm the versatility of the SA metabolism in response to different pathogens.

These specific differences in tomato SA metabolism generated by both pathogens could also be explained by SA bacterial metabolism itself. Whilst CEVd is a non-coding pathogen totally dependent on host transcriptional machinery, *Pst* possesses its own SA biosynthetic pathway which is different from plants [62]. Moreover, plant SA accumulation is a target of suppression by *Pst* [63], and the bacterial wilt pathogen *Ralstonia solanacearum* has been described to degrade plant SA in order to protect itself from inhibitory levels of this compound as well as to enhance its virulence on plant hosts [64]. Therefore, while the observed SA metabolism caused by CEVd infection is totally plant-dependent, the participation of the *Pst* SA metabolism could in part explain the differences observed in *Pst*-infected *RNAi_SlS5H* tomato plants, including the lack of SA accumulation.

The role of SA on leaf senescence has long been described [65]. In this sense, impairment of S3H or S5H in Arabidopsis provokes an advanced senescent response [21, 23]. However, no distinctive phenotypic differences regarding senescence were reported for *SlDMR6-1* tomato mutant [24]. Here we have studied the effect of *SlS5H* silencing on the development of tomato plants, observing an early senescence phenotype provoked by SA over-accumulation (Fig. 9). These differences could be due to the tomato variety employed for the studies, since Fla. 8000 was used by Thomazella et al. [24] and Moneymaker tomato plants were used in our studies. Additionally, the differences between CRISPR knock out and RNAi silencing approaches could also be considered. According to our results, a higher induction of both *SAG12* and *NOR* senescence markers [46], as well as *PR1*, a molecular marker for the defence response [66], was detected in both transgenic plants silencing *SlS5H* (Fig. 9). As previously stated, the toxic effect of SA over-accumulation, also responsible for the observed cell death and the decrease in chlorophyll, could be related with ROS accumulation [59]. Our results provide genetic evidence that *SlS5H* has an important role in regulating the onset and rate of leaf senescence in tomato by fine-tuning the endogenous levels of SA.

## Conclusion

Through the balance between *de novo* biosynthesis, catabolism and reversible deactivation, plants manage to maintain their endogenous levels of SA. Hydroxylation of SA to produce GA is performed by S5H, which has been described as DMR6 in Arabidopsis [23] and tomato [24]. Infections with CEVd and *Pst* produce high and low levels of GA, respectively [33, 36–38], thus constituting excellent models to study the role of S5H in tomato plants. Here we demonstrate that the impairment of SA hydroxylation increases resistance to CEVd and *Pst* in tomato. Surprisingly, the observed resistance is accompanied by an increase in SA levels in* RNAi_SlS5H* plants only upon CEVd, but not upon *Pst* infection, where SA appeared to be rerouted to other phenolics involved in defence. Our results indicate there is an additional complexity level associated to pathogen-induced specific SA homeostasis, suggesting that the framework of SA biology, which is established mainly for *Pst*-infected *Arabidopsis thaliana* plants, needs to be considered for each specific plant-pathogen interaction.

## Methods

### Plant materials and growth conditions

Tomato Rio Grande plants, containing the *Pto* resistance gene (gently provided by Dr. Selena Giménez, Centro Nacional de Biotecnología, Madrid, Spain), were used to establish the virulent and avirulent interaction upon bacterial infections. In the rest of experiments, transgenic tomato (*Solanum lycopersicum*) plants silencing the endogenous salicylate 5-hydroxylase gene (*SlS5H*) and the cultivar Moneymaker (gently provided by Dr. Prof. Jonathan Jones, The Sainsbury Laboratory, Norwich, UK), the isogenic parental line of *RNAi_SlS5H*, were used.

Tomato seeds were surface sterilized with sodium hypochlorite. After sterilization, seeds were sown in 12 cm-diameter pots and grown in standard greenhouse conditions, with a temperature between 25 and 30 °C, a relative humidity of 50–70% and long day photoperiod (16 h light/8 h darkness).

For transient expression experiments, *Nicotiana benthamiana* plants were cultivated in the same conditions as tomato plants.

### Vector construction

The full-length cDNA (1014 bp) of salicylate 5-hydroxylase gene (*SlS5H;* Solyc03g080190*)* was amplified by RT-PCR from leaves of Moneymaker tomato plants infected with CEVd, 4 weeks after inoculation, using 5′ ATGGAAACCAAAGTTATTTC-3′ as the forward primer and 5′-GTTCTTGAAAAGTTCCAAAC-3′ as the reverse primer. The resulting PCR product was cloned into the pCR8/GW/TOPO entry vector (Invitrogen), following the manufacturer’s protocol, and was sequenced. Then *SlS5H* was subcloned in the pGWB8 Gateway binary vector [67]. This vector carries the CaMV35S promoter and the hexahistidine tag (6XHis) which is attached to the C-terminus of the recombinant protein.

In order to generate the *SlS5H*-silenced transgenic tomato plants, the method described by Helliwell and Waterhouse was followed [68]. Briefly, a selected 400 bp sequence of *SlS5H* was amplified from the full-length cDNA clone using the forward primer 5′-GGCTCGAGTCTAGAGGGAAATTCGTCAA-3′, which introduced restriction sites *Xho*I and *Xba*I, and the reverse primer 5′-CCGAATTCGGATCCACCGTTACTTTACTGC-3′, which added restriction sites *BamH*I and *EcoR*I. The PCR product was first cloned in the pGEM T Easy vector (Promega) and sequenced. After digestion with the appropriate restriction enzymes and purification, the two *SlS5H* fragments were subcloned into the pHANNIBAL vector in both the sense and the antisense orientations. Finally, the constructs made in pHANNIBAL were subcloned as a *Not*I flanked fragment into pART27 binary vector to produce highly effective intron-containing “hairpin” RNA silencing constructs [69]. This vector carries the neomycin phosphotransferase gene (*NPT II*) as a transgenic selectable marker.

### *Nicotiana benthamiana* agroinfiltration and tomato transformation

The *pGWB8-SlS5H *construction and the *pGWB8* empty vector were transformed into the *Agrobacterium tumefaciens* C58 strain, while the *pART27-SlS5H* construction was transformed into *A. tumefaciens* LBA4404. Leaves of 4-week-old *N. benthamiana* plants were infiltrated with the *A. tumefaciens* C58 strain carrying *pGWB8-SlS5H* or the empty vector, along with a 1:1 ratio of the C58 strain carrying the p19 plasmid, which encodes the silencing suppressor protein p19 [70]. Tomato Moneymaker cotyledons were co-cultured with *A. tumefaciens* LBA4404 carrying the *pART27-SlS5H* construction to generate the RNAi *SlS5H*-silenced transgenic tomato plants (*RNAi_SlS5H*). The explant preparation, selection and regeneration methods followed those published by Ellul and co-workers [71]. The tomato transformants were selected in kanamycin-containing medium and propagated in soil. Moneymaker wild-type tomato plants regenerated in vitro from cotyledons under the same conditions as the transgenic lines were used as controls in subsequent analyses. The transgenic plants generated in this study have been produced, identified and characterized in our laboratory and are to be used exclusively for research purposes.

### Production of S5H recombinant protein in *Nicotiana benthamiana*

Agroinfiltration of *N. benthamiana* leaves with *Agrobacterium tumefaciens* C58, carrying either *pGWB8-SlS5H* or *pGWB8 *empty vector was performed according to Yang et al. [72]. Three days after the agro-inoculation, plants were embedded in a solution of 1 mM SA (see SA treatments) and samples were collected 24 upon the treatment. For western blot analysis, 5 g of frozen agroinfiltrated *N. benthamiana* leaves were ground and resuspended in 1 mL of extraction buffer (50 mM Tris-HCl, pH 7.5, containing 15 mM 2-mercaptoethanol). Proteins were separated by SDS-PAGE and stained with Coomassie Brilliant Blue R-250 [73] or transferred to nitrocellulose filters (OPTITRAN, Schleicher&Schuell) following the protocol described by Towbin et al. [74]. S5H recombinant protein was examined by using specific anti-His mouse antibody (Novagen) as described in [36].

### SA treatments

The SA treatments were carried out by stem-feeding [75]. Four-week-old tomato plants or agro-inoculated *N. benthamiana* plants were excised above cotyledons, and stem cuts were immediately immersed in a 2 mM or 1 mM SA solution, respectively. After 30 min, all the stems were transferred to water, and leaf samples from three biological replicates were taken at 0, 0.5, 1, 6, 24 and 48 h post-treatment.

### Viroid inoculation

For these assays, tomato plants were grown on a growth chamber with a temperature between 28 °C/24°C and a relative humidity of 60%/85% (day/night). Viroidal inoculum was prepared from leaves of CEVd-infected tomato plants as previously described [76] and the first cotyledon and leaf of each plant were inoculated using carborundum as abrasive. Mock plants were inoculated with sterile water. Disease and symptom severity was recorded periodically. Leaf samples from six infected or mock-inoculated plants were harvested at 2- and 3-weeks post inoculation (wpi).

### *Pseudomonas syringae* inoculation

The bacterial strain used in this study was *Pseudomonas syringae* pv. *tomato* DC3000 (*Pst*). For incompatible interaction, the infection was performed by the bacterial strain Pst DC3000 that contains deletions in genes *avrPto* and *avrPtoB* (Pst DC3000 ΔavrPto/ΔavrPtoB) [77].

Pathogen inoculation was performed in 4-week-old tomato plants by immersion, as previously described [78]. For mock treatments, plants were immersed in 10 mM sterile MgCl_2_ containing 0.05% Silwet L-77. The third and fourth leaves from six plants per treatment and genotype were harvested 24 h after inoculation.

### *Botrytis cinerea* inoculation assays

The *B. cinerea* strain used was CECT2100 (Spanish Type Culture Collection). Fungal hyphae were grown on potato dextrose agar for 14 days at 24 °C in darkness. Spore suspensions were prepared by scraping surface plates, washing with sterile water, and filtering through cotton. Finally, the concentration was adjusted to 10^6^ spores/mL. Three leaflets per plant were spotted with a 5 mL droplet of the spore suspension. All the experiments were carried out into inoculation chambers to maintain the proper high humidity conditions. Photographs, lesion size measurements and sampling were performed at 5 days after inoculation.

### Determination of chlorophyll content

Chlorophyll quantification was carried out by the method of Arnon [79]. Frozen leaf tissue was homogenized with 80% acetone and then incubated overnight at 4 °C. Then, samples were centrifuged and the absorbance of the extracted solution was measured at 645 and 663 nm. Determination of chlorophyll a, b and total concentration was determined according to Arnon’s equations.

### Extraction and HPLC analysis of salicylic and gentisic acids

Extraction of free and total SA and GA from tomato leaflets was performed according to our previously published protocol [34]. Aliquots of 30 µL were injected through a Waters 717 autosampler into a reverse-phase Sun Fire 5-mm C18 column (4.6 mm x 150 mm) equilibrated in 1% (v/v) acetic acid at room temperature. A 20-min linear gradient of 1% acetic acid to 100% methanol was applied using a 1525 Waters Binary HPLC pump at a flow rate of 1 mL/min. SA and GA were detected with a 2475 Waters Multi-l Fluorescence detector (λ excitation 313 nm; λ emission 405 nm) and were quantified with the Waters Empower Pro software using authentic standard compounds (SA sodium salt and GA, Sigma–Aldrich, Madrid, Spain). Standard curves were performed for each compound using similar concentration ranges to those detected in the samples. Data were corrected for losses in the extraction procedure, and recovery of metabolites ranged between 50 and 80%.

### UPLC-ESI-QTOF-MS analysis

For UPLC-MS analysis, frozen tomato leaves (100 mg) were ground into powder in liquid nitrogen and extracted in 1 mL of methanol/water (80:20, v/v) for chromatographic analysis.

UPLC separations were performed on a reverse phase Poroshell 120 EC-C18 column (3 × 100 mm, 2.7 μm) (Agilent Technologies) operating at 30 °C and a flow rate of 0.4 mL/min. The mobile phases used were acidified water (0.1% formic acid) (Phase A) and acidified acetonitrile (0.1% formic acid) (Phase B). Compounds were separated using the following gradient conditions: 0–10 min, 1–18% phase-B; 10–16 min, 18–38% phase- B; 16–22 min, 38–95% phase-B. Finally, the phase B content was returned to the initial conditions (1% phase-B) for 1 min and the column re-equilibrated for five minutes more. 7 µL of the sample was injected using flow through needle (FTN) injection with a 15 mL syringe. The sample compartment in the auto sampler was maintained at 7.0 °C.

The UPLC system was coupled to a quadrupole-time-of-flight (maXis Impact HR Q-ToF-MS, (Bruker Daltonik GmbH, Bremen, Germany) orthogonal accelerated Q-ToF mass spectrometer, was performed using HR-ToF-MS in negative electrospray ionization mode using broadband collision induced dissociation (bbCID). High and low collision energy data were collected simultaneously by alternating the acquisition between MS and bbCID conditions.

Parameters for analysis were set using negative ion mode, with spectra acquired over a mass range from 50 to 1200 *m/z*. The optimum values of the ESI-MS parameters were: capillary voltage, -4.0 kV; drying gas temperature, 200 °C; drying gas flow, 9.0 L/min; nebulising gas pressure, 2 bars; collision RF, 150 Vpp; transfer time 72 µs, and pre-pulse storage, 5 µs.

At some stage in the UHPLC method development, an external apparatus calibration was performed using a KD Scientific syringe pump (Vernon Hills, IL) directly linked to the interface, passing a solution of sodium formate with a flow rate of 180 µL/h. The instrument was calibrated externally before each sequence with a 10 mM sodium formate solution.

Using this method, an exact calibration curve based on numerous cluster masses each differing by 68 Da (CHO_2_Na) was obtained. Due to the compensation of temperature drift in the Q-TOF, this external calibration provided accurate mass values for a complete run without the need for a dual sprayer set up for internal mass calibration.

### Non-targeted metabolomics analysis and quality control

All samples were injected in the same batch and the order of sample injection was randomized to avoid sample bias. A mixture with one replicate of each group of samples was used as ‘‘quality control’’ (QC) and was injected at the beginning, in the middle and at the end of the batch. Besides, methanol/water injections were included every five samples as a blank run to avoid the carry-over effect.

For the untargeted analysis of the polar and semi-polar profiles, the QToF-MS data were processed with XCMS online resources (https://xcmsonline.scripps.edu) with the appropriate script for the alignment of chromatograms and the quantification of each MS feature [80]. The resulting dataset was submitted to a Principal Component Analysis (PCA) study by the SIMCA-P software (v. 11.0, Umetrics, Umeå, Sweden) using unit variance (UV) scaling.

Metabolite identification was based on comparison of accurate mass, retention time, MS/MS fragments and CCS values with online reference databases including Respect (https://spectra.psc.riken.jp/), Metlin (https://metlin.scripps.edu/), HMDB (https://hmdb.ca/), Lipidmap (https://www.lipidmaps.org/), in-house databases based on commercial standards and theoretical MS/MS tags, and bibliographies. The CCS value acceptable error was < 5% with MS tolerance of 5 p.p.m., and MS/MS tolerance of < 10 mDa, at least one major fragment was found.

### RNA extraction and quantitative RT-PCR analysis

The total RNA of tomato leaves was extracted using the TRIzol reagent (Invitrogen, Carlsbad, CA, United States), following the manufacturer’s protocol. RNA was then precipitated by adding one volume of 6 M LiCl and keeping it on ice for 4 h. Afterward the pellet was washed using 3 M LiCl and was dissolved in RNase-free water. Finally, to remove any contaminating genomic DNA, 2 U of TURBO DNase (Ambion, Austin, TX, United States) were added per microliter of RNA. For the quantitative RT-PCR (qRT-PCR) analysis, one microgram of total RNA was employed to obtain the corresponding cDNA target sequences using an oligo(dT)18 primer and the PrimeScript RT reagent kit (Perfect Real Time, Takara Bio Inc., Otsu, Shiga, Japan), following the manufacturer’s directions. Quantitative PCR was carried out as previously described [81]. A housekeeping gene transcript, actin or elongation factor 2, was used as the endogenous reference. The PCR primers were designed using the online service Primer3 (https://primer3.ut.ee/) and are listed in Table S1.

### Statistical analysis

The statistical analysis of two or more variables was carried out by using Student’s *t*-test or analysis of variance (ANOVA), respectively, employing the Prism 9 software (https://www.graphpad.com/). Tukey’s post hoc tests were performed in ANOVA analyses. In all the analyses, a *p*-value < 0.05 was considered statistically significant.

## Supplementary Information

**Additional file 1:** **Figure S1.** Phylogenetic analysis of*AtS5H* orthologs in tomato. The box in the phylogenetic tree highlights *AtS5H* from *Arabidopsis thaliana* (At5g24530) and its closest homolog in tomato (Solyc03g080190). The multiple alignment was made using ClustalW and the dendrogram was built using the MegAlign program from the Lasergene package (DNASTAR, Madison, Wisconsin, USA). **Figure S2.** SA-induced expression of *SlS5H* in wild type (WT) tomato plants.*SlS5H* (A) and *PR1*(B) expression of tomato plants treated with 2 mM of SA (SA) or water (MOCK) by stem feeding at 0, 0.5, 1, 6, 24 and 48 h post-treatment. The qRT-PCR values were normalized with the level of expression of the actin gene. The expression levels correspond to the mean ± the standard error of a representative experiment (*n* = 3). Significant differences between mock and infected or treated plants at different time points are represented by different letters when *p*-value < 0.05. No statistical differences were observed regarding *SlS5H* gene expression. **Figure S3.** S5H *in vivo* activity in *Nicotiana benthamiana* plants. (A) SDS-PAGE (left panel) and western blot analysis (right panel) of *N. benthamiana* plants agroinoculated with pGWB8 empty vector (C) or pGWB8-SlS5H (S5H). (B) Diagram of the cloning cassette. Panels on the right show the nanomoles of SA (C) and GA (D) per gram of fresh weight in *Nicotiana benthamiana* leaves embedded with SA and agroinoculated with the construction pGWB8-S5H, compared with its control (plasmid pGWB8 without insert). The results correspond to a representative experiment (*n* = 3). Student’s *t*-statistic analysis shows the mean ± standard deviation since *p*-value < 0.001 in free (**) and total (**’) SA accumulation. No statistical differences were observed for GA accumulation. 2,3-DHBA was not detected. **Figure S4.** Gene expression analysis of wild type (WT) and *RNAi_SlS5H*(lines 14 and 16) transgenic tomato plants, mock-inoculated (MOCK) and inoculated with CEVd (CEVd). *DCL1*(A), *DCL2*(B), *RDR1*(C) and *TCI21*(D) gene expression was analyzed 3 weeks after viroid infection. The qRT-PCR values were normalized with the level of expression of the actin gene. The expression levels correspond to the mean ± the standard error of a representative experiment (*n* = 3). The significant differences between different genotypes and infected or mock-inoculated plants are represented by different letters since *p*-value < 0.05. **Figure S5.** Score plot of PCA based on whole range of on the whole array of the mass spectra within a *m/z* range from 100 to 1500 using unit variance (UV) scaling method of methanolic extracts from tomato leaves. (A) CEVd infected plants at 3 wpi, green: wild type (WT); light purple: *RNAi_SlS5H* 14; dark purple: *RNAi_SlS5H* 16; (B) *Pst* infected plants at 24 hpi, yellow: wild type (WT); orange: *RNAi_SlS5H* 14; brown: *RNAi_SlS5H* 16. **Figure S6.** Analysis of phenotypic differences between WT and*RNAi*_S5H transgenic lines 14 and 16. Differences related to weight (A), internode length (B), conductivity (C) and chlorophyll content (D) in WT and *RNAi_SlS5H* 14 and 16 transgenic plants were measured 10 weeks after germination. Bars represent the mean ± the standard deviation of a representative experiment (*n* = 6)

## Data Availability

Material will be available upon request to authors after MTA signature. The datasets generated and/or analysed during the current study are not publicly available since they are stored in our local computers and are available from the corresponding author on reasonable request. The database links mentioned in the manuscript are listed below: https://solgenomics.net/. www.arabidopsis.org. http://plants.ensembl.org/index.html. https://xcmsonline.scripps.edu. https://spectra.psc.riken.jp/. https://metlin.scripps.edu/. https://hmdb.ca/. https://www.lipidmaps.org/. https://primer3.ut.ee/. https://www.graphpad.com.
