# Supplementary material for: SlS5H silencing reveals specific pathogen-triggered salicylic acid metabolism in tomato
Source: BMC Plant Biol. 2022 Nov 29;22:549. doi: 10.1186/s12870-022-03939-5 (PMC9706870; doi:10.1186/s12870-022-03939-5)

Figure S1

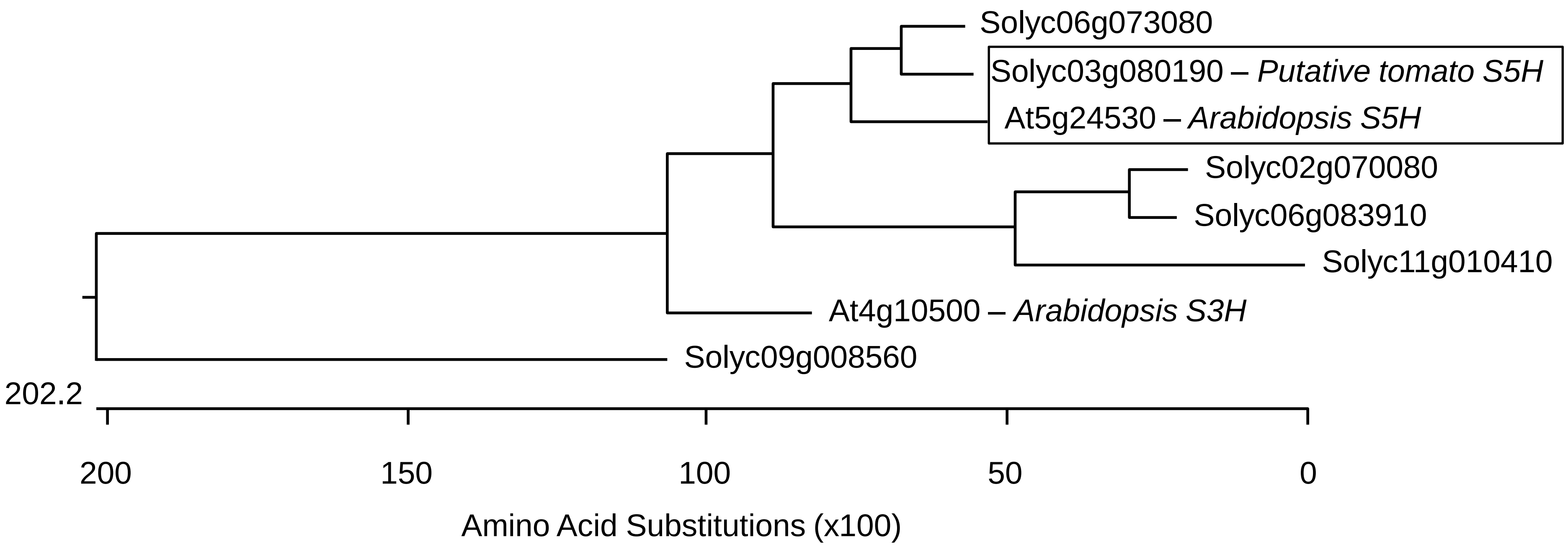

Figure S1. Phylogenetic analysis of *AtS5H* orthologs in tomato. The box in the phylogenetic tree highlights *AtS5H* from *Arabidopsis thaliana* (At5g24530) and its closest homolog in tomato (Solyc03g080190). The multiple alignment was made using ClustalW and the dendrogram was built using the MegAlign program from the Lasergene package (DNASTAR, Madison, Wisconsin, USA).

Figure S2

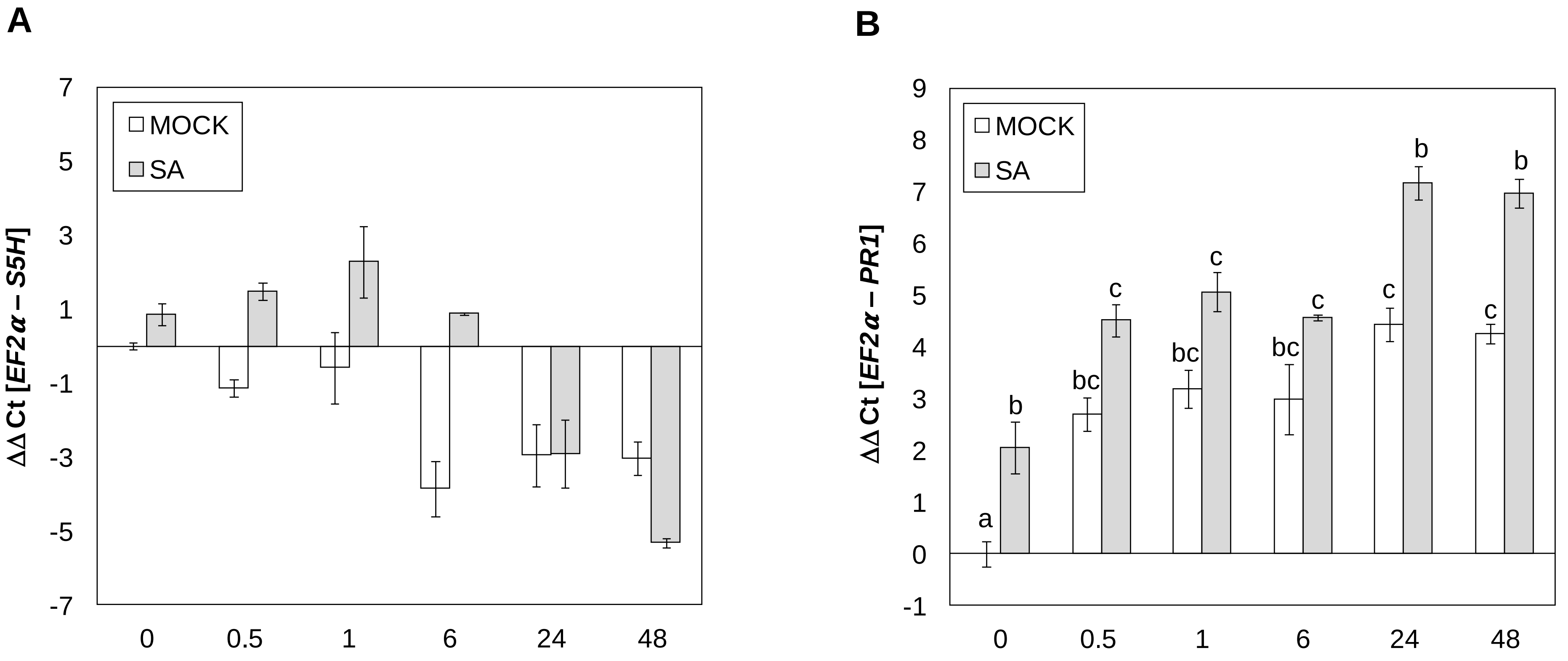

Figure S2. SA-induced expression of *SlS5H* in wild type (WT) tomato plants. *SlS5H* **(A)** and *PR1* **(B)** expression of tomato plants treated with 2 mM of SA (SA) or water (MOCK) by stem feeding at 0, 0.5, 1, 6, 24 and 48 hours post-treatment. The qRT-PCR values were normalized with the level of expression of the actin gene. The expression levels correspond to the mean ± the standard error of a representative experiment (n=3). Significant differences between mock and infected or treated plants at different time points are represented by different letters when p-value < 0.05. No statistical differences were observed regarding *SlS5H* gene expression.

Figure S3

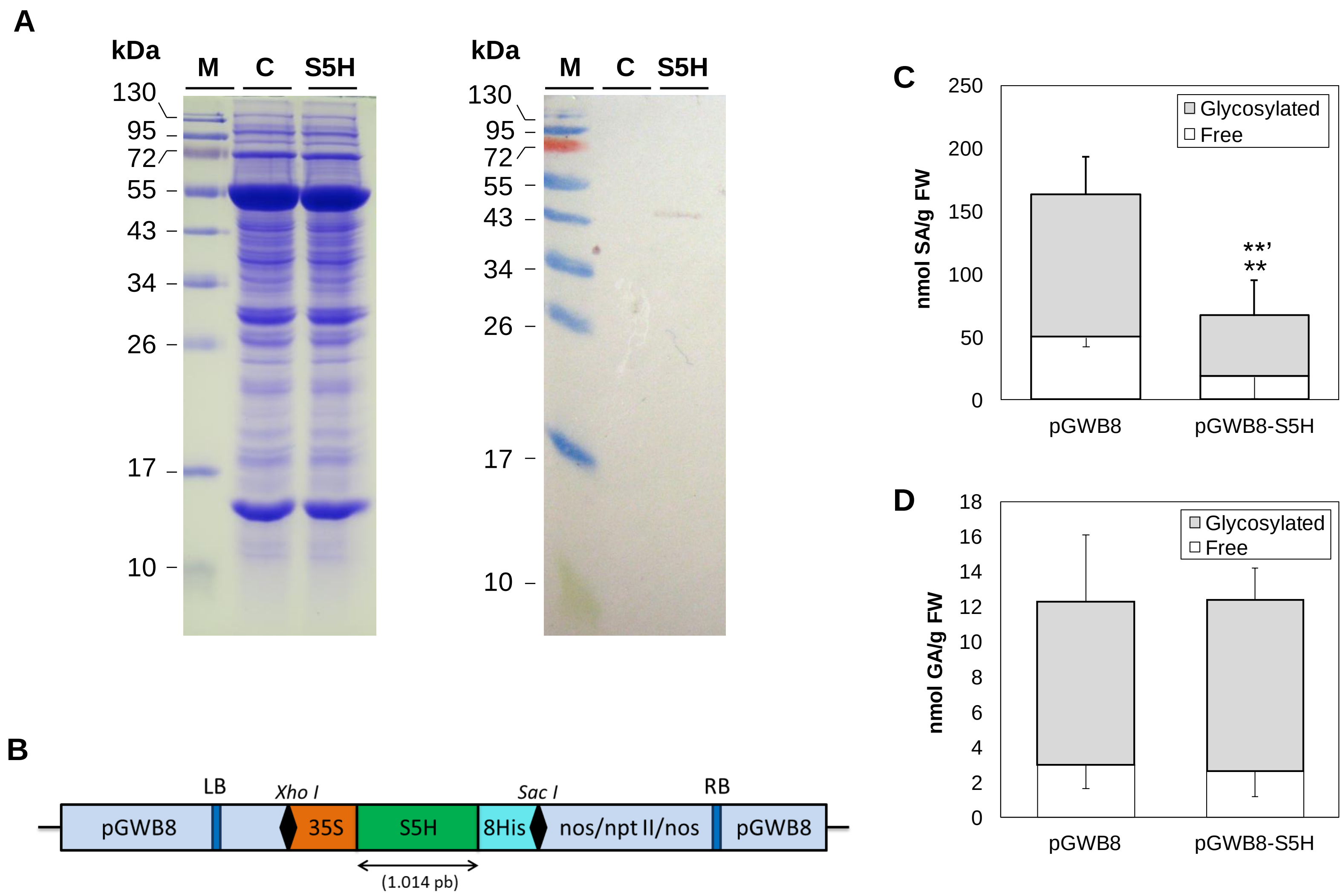

Figure S3. S5H *in vivo* activity in *Nicotiana benthamiana* plants. (A) SDS-PAGE (left panel) and western blot analysis (right panel) of *N. benthamiana* plants agroinoculated with pGWB8 empty vector (C) or pGWB8-SlS5H (S5H). **(B)** Diagram of the cloning cassette. Panels on the right show the nanomoles of SA **(C)** and GA **(D)** per gram of fresh weight in *Nicotiana benthamiana* leaves embedded with SA and agroinoculated with the construction pGWB8-S5H, compared with its control (plasmid pGWB8 without insert). The results correspond to a representative experiment (n=3). Student's *t*-statistic analysis shows the mean ± standard deviation since *p*-value < 0.001 in free (**) and total (**') SA accumulation. No statistical differences were observed for GA accumulation. 2,3-DHBA was not detected.

Figure S4

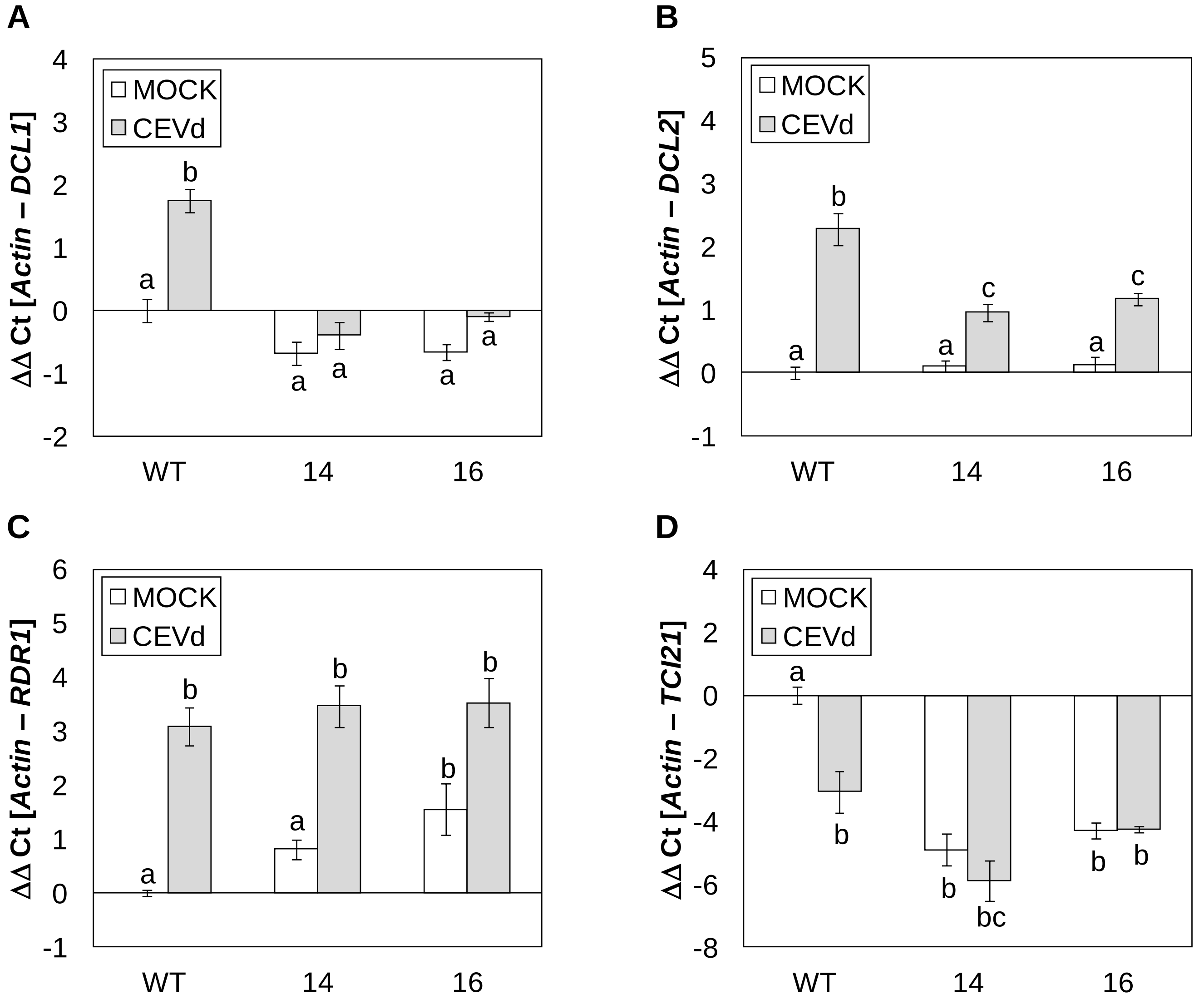

Figure S4. Gene expression analysis of wild type (WT) and *RNAi_SlS5H* (lines 14 and 16) transgenic tomato plants, mock-inoculated (MOCK) and inoculated with CEVd (CEVd). *DCL1* **(A)**, *DCL2* **(B),** *RDR1* **(C)** and *TCl21* **(D)** gene expression was analyzed 3 weeks after viroid infection. The qRT-PCR values were normalized with the level of expression of the actin gene. The expression levels correspond to the mean ± the standard error of a representative experiment (n=3). The significant differences between different genotypes and infected or mock-inoculated plants are represented by different letters since p-value < 0.05.

Figure S5

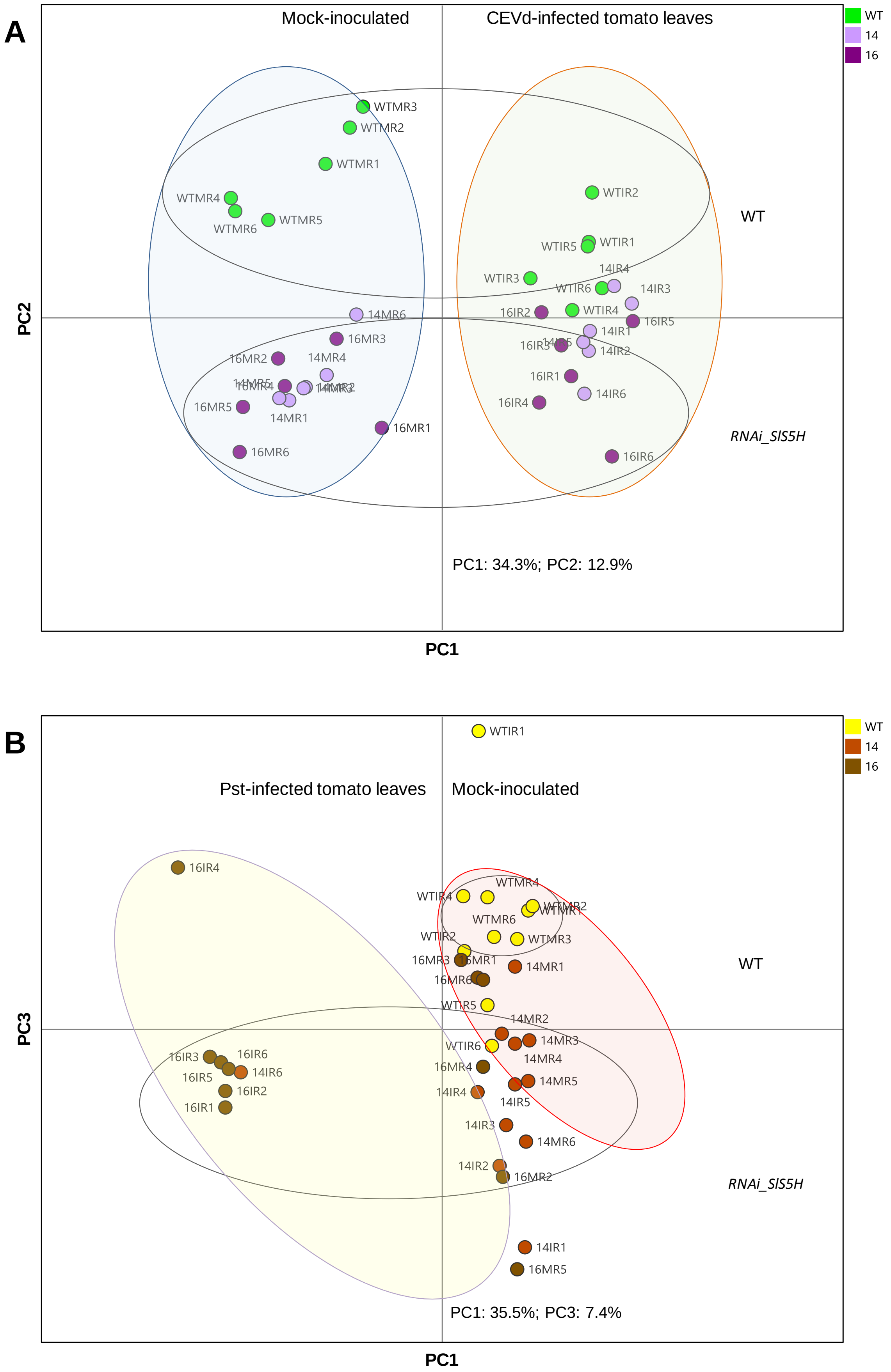

Figure S5. Score plot of PCA based on whole range of on the whole array of the mass spectra within a *m/z* range from 100 to 1500 using unit variance (UV) scaling method of methanolic extracts from tomato leaves. (A) CEVd infected plants at 3 wpi, green: wild type (WT); light purple: *RNAi_SlS5H* 14; dark purple: *RNAi_SlS5H* 16; **(B)** *Pst* infected plants at 24 hpi, yellow: wild type (WT); orange: *RNAi_SlS5H* 14; brown: *RNAi_SlS5H* 16.

Figure S6

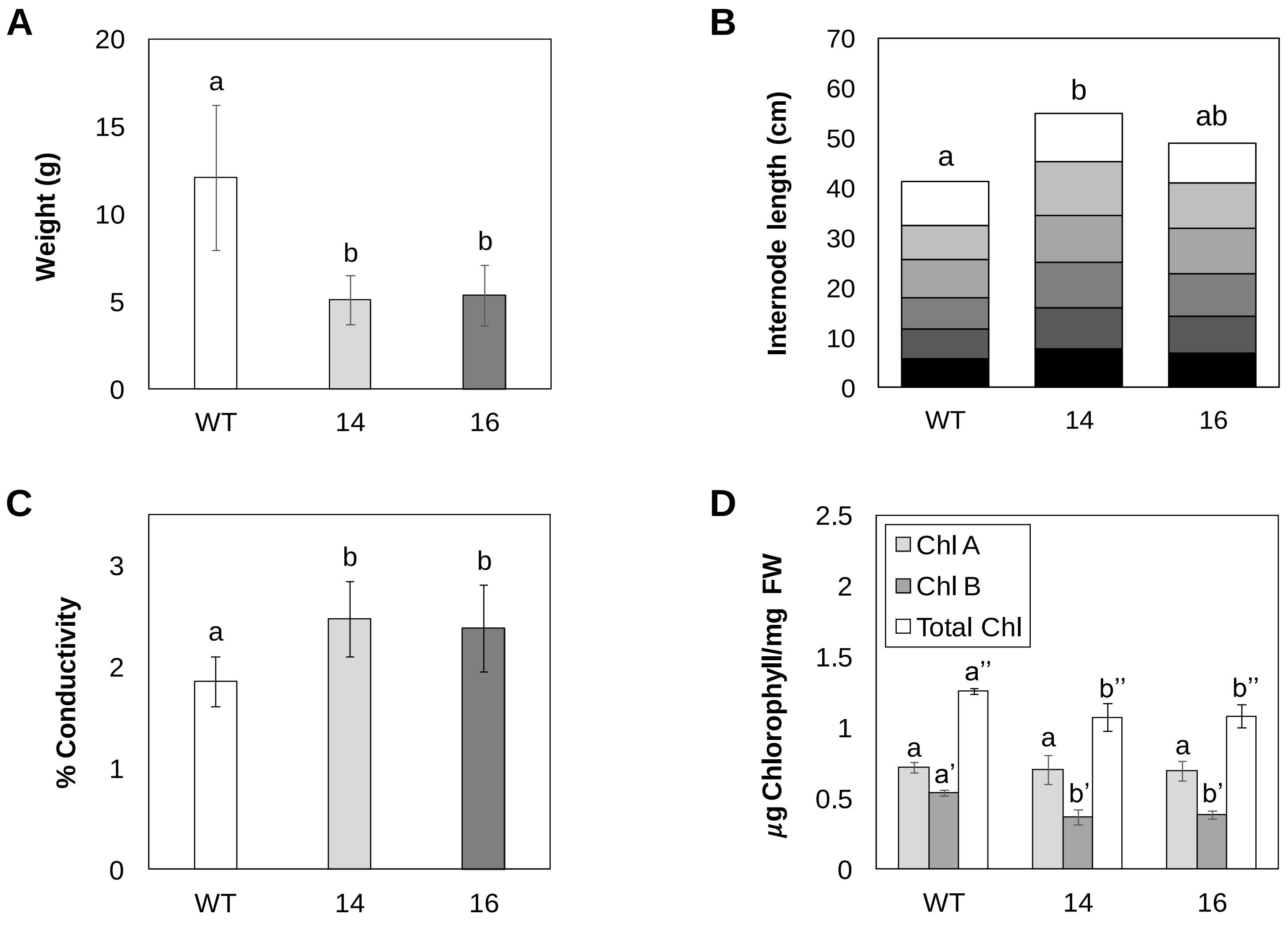

Figure S6. Analysis of phenotypic differences between WT and *RNAi*_S5H transgenic lines 14 and 16. Differences related to weight **(A)**, internode length **(B)**, conductivity **(C)** and chlorophyll content **(D)** in WT and *RNAi_SlS5H* 14 and 16 transgenic plants were measured 10 weeks after germination. Bars represent the mean ± the standard deviation of a representative experiment (n=6).

Original snapshots for Supplemental Figure S3. Pictures were taken with a Canon PowerShot A650-IS at a resolution of 4000 x 3000 pixels in AUTO mode, macro and autofocus activated. Tracks used in Figure S3 are indicated.

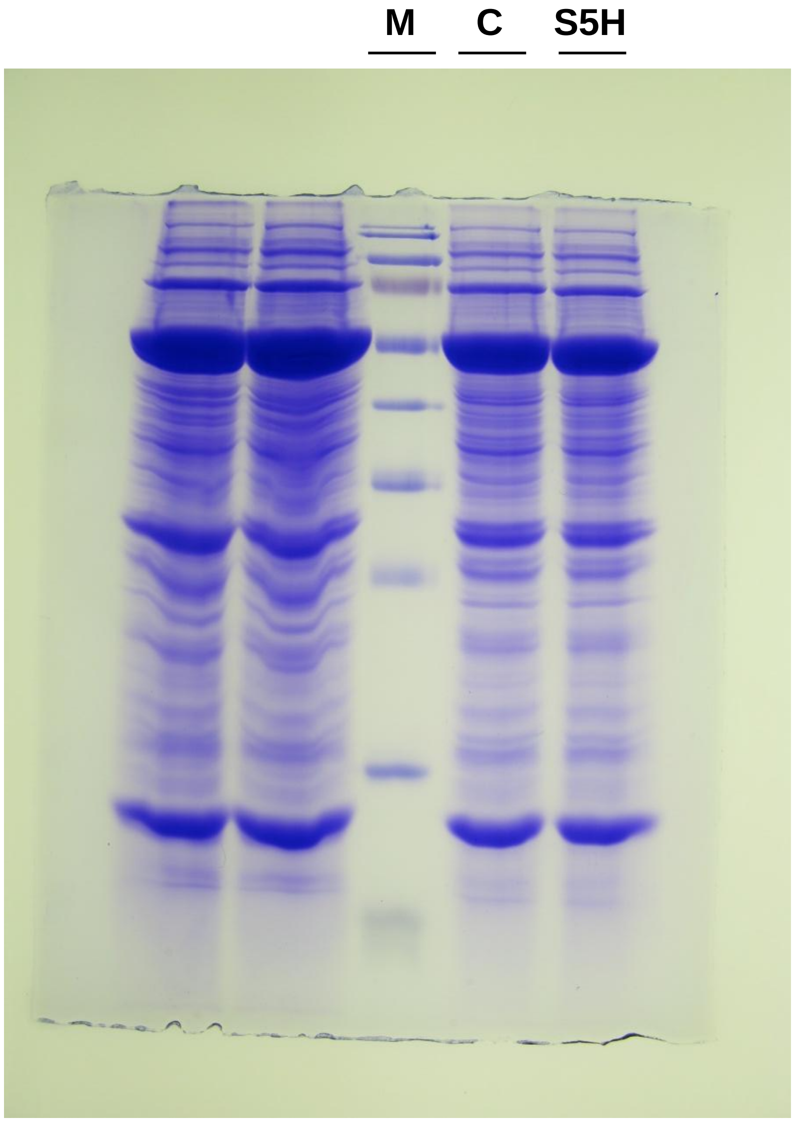

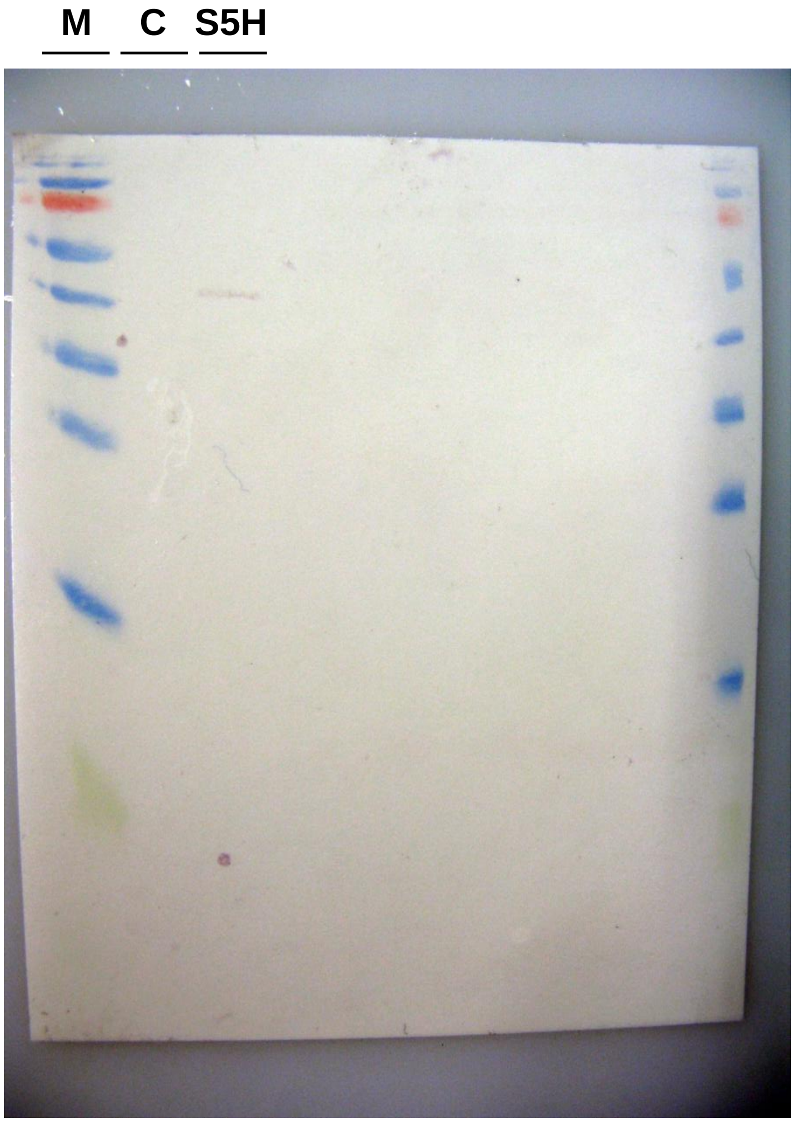

Supplement: Supplementary file 1 — Additional file 1: Figure S1. Phylogenetic analysis ofAtS5H orthologs in tomato. The box in the phylogenetic tree highlights AtS5H from Arabidopsis thaliana (At5g24530) and its closest homolog in tomato (Solyc03g080190). The multiple alignment was made using ClustalW and the dendrogram was built using the MegAlign program from the Lasergene package (DNASTAR, Madison, Wisconsin, USA). Figure S2. SA-induced expression of SlS5H in wild type (WT) tomato plants.SlS5H (A) and PR1(B) expression of tomato plants treated with 2 mM of SA (SA) or water (MOCK) by stem feeding at 0, 0.5, 1, 6, 24 and 48 h post-treatment. The qRT-PCR values were normalized with the level of expression of the actin gene. The expression levels correspond to the mean ± the standard error of a representative experiment (n = 3). Significant differences between mock and infected or treated plants at different time points are represented by different letters when p-value < 0.05. No statistical differences were observed regarding SlS5H gene expression. Figure S3. S5H in vivo activity in Nicotiana benthamiana plants. (A) SDS-PAGE (left panel) and western blot analysis (right panel) of N. benthamiana plants agroinoculated with pGWB8 empty vector (C) or pGWB8-SlS5H (S5H). (B) Diagram of the cloning cassette. Panels on the right show the nanomoles of SA (C) and GA (D) per gram of fresh weight in Nicotiana benthamiana leaves embedded with SA and agroinoculated with the construction pGWB8-S5H, compared with its control (plasmid pGWB8 without insert). The results correspond to a representative experiment (n = 3). Student’s t-statistic analysis shows the mean ± standard deviation since p-value < 0.001 in free (**) and total (**’) SA accumulation. No statistical differences were observed for GA accumulation. 2,3-DHBA was not detected. Figure S4. Gene expression analysis of wild type (WT) and RNAi_SlS5H(lines 14 and 16) transgenic tomato plants, mock-inoculated (MOCK) and inoculated with CEVd (CEVd). DCL1(A), DCL2 [file 12870_2022_3939_MOESM1_ESM.pdf]
